# High-throughput mediation analysis of human proteome and metabolome identifies mediators of post-bariatric surgical diabetes control

**DOI:** 10.1038/s41467-021-27289-2

**Published:** 2021-11-29

**Authors:** Jonathan M. Dreyfuss, Yixing Yuchi, Xuehong Dong, Vissarion Efthymiou, Hui Pan, Donald C. Simonson, Ashley Vernon, Florencia Halperin, Pratik Aryal, Anish Konkar, Yinong Sebastian, Brandon W. Higgs, Joseph Grimsby, Cristina M. Rondinone, Simon Kasif, Barbara B. Kahn, Kathleen Foster, Randy Seeley, Allison Goldfine, Vera Djordjilović, Mary Elizabeth Patti

**Affiliations:** 1 Bioinformatics and Biostatistics Core, Research Division, Joslin Diabetes Center, Boston, MA USA; 2 Biomedical Engineering, Boston University, Boston, MA USA; 3 Harvard Medical School, Boston, MA USA; 4 Integrative Physiology and Metabolism, Research Division, Joslin Diabetes Center, Boston, MA USA; 5 Department of Medicine, Brigham and Women’s Hospital, Boston, MA USA; 6 Department of Surgery, Brigham and Women’s Hospital, Boston, MA USA; 7 Division of Endocrinology, Diabetes & Metabolism, Department of Medicine, Beth Israel Deaconess Medical Center, Boston, MA USA; 8 MedImmune, Gaithersburg, MD USA; 9 Department of Surgery, University of Michigan, Ann Arbor, MI USA; 10 Department of Economics, Ca’ Foscari University of Venice, Venice, Italy; 11 Present Address: Vertex Pharmaceuticals, Boston, MA USA; 12 Present Address: Department of Endocrinology, Diabetes & Metabolism, Sir Run Run Shaw Hospital, Zhejiang University School of Medicine, Hangzhou, China; 13 Present Address: Form Health, Boston, MA USA; 14 Present Address: Eli Lilly and Company, Indianapolis, IN USA; 15 Present Address: Genmab, Plainsboro, NJ USA; 16 Present Address: AstraZeneca, Gaithersburg, MD USA; 17 Present Address: Novartis Institute for Biomedical Research, Cambridge, MA USA

**Keywords:** Statistical methods, Type 2 diabetes

## Abstract

To improve the power of mediation in high-throughput studies, here we introduce High-throughput mediation analysis (Hitman), which accounts for direction of mediation and applies empirical Bayesian linear modeling. We apply Hitman in a retrospective, exploratory analysis of the SLIMM-T2D clinical trial in which participants with type 2 diabetes were randomized to Roux-en-Y gastric bypass (RYGB) or nonsurgical diabetes/weight management, and fasting plasma proteome and metabolome were assayed up to 3 years. RYGB caused greater improvement in HbA1c, which was mediated by growth hormone receptor (GHR). GHR’s mediation is more significant than clinical mediators, including BMI. GHR decreases at 3 months postoperatively alongside increased insulin-like growth factor binding proteins IGFBP1/BP2; plasma GH increased at 1 year. Experimental validation indicates (1) hepatic GHR expression decreases in post-bariatric rats; (2) GHR knockdown in primary hepatocytes decreases gluconeogenic gene expression and glucose production. Thus, RYGB may induce resistance to diabetogenic effects of GH signaling.

Trial Registration: Clinicaltrials.gov NCT01073020.

## Abstract (web summary)

Factors underlying the effects of gastric bypass surgery on glucose homeostasis are incompletely understood. Here the authors developed and applied high-throughput mediation analysis to identify proteome/metabolome mediators of improved glucose homeostasis after to gastric bypass surgery, and report that improved glycemia was mediated by the growth hormone receptor.

## Introduction

Given the major public health and personal burden of T2D, the identification of new approaches to therapy for both T2D and obesity is critical. Increasing evidence supports that surgical approaches to T2D are effective for both long-term weight loss and improved glucose metabolism, resulting in remission of T2D in ≈90% of patients at 1 year and 45% at 5 years^1–5^, and that these effects are superior to traditional nonsurgical diabetes management. Surgery’s beneficial effects have been attributed to weight loss and improved insulin sensitivity, but improved glycemic control occurs within days after RYGB, before substantial weight loss, supporting an important role for weight-independent mechanisms^6^. Additional metabolic effects of surgery which may contribute to improved glycemic outcomes include increased postprandial insulin and incretin hormone secretion^7,8^, reduction in circulating amino acids^9,10^, alterations in bile acids, FXR signaling and FGF19^11–15^, and changes in microbiome metabolism^16–18^, but preclinical studies indicate these cannot fully explain clinical improvement^19–21^. Thus, the primary molecular factors mediating improved metabolism and remission of T2D in response to surgical procedures remain uncertain.

To identify mediators of metabolic improvement after surgical vs. medical therapy for T2D, we perform a retrospective exploratory analysis of the SLIMM-T2D trial, in which participants with type 2 diabetes were randomized to Roux-en-Y gastric bypass (RYGB) or nonsurgical diabetes weight management (DWM), and fasting plasma proteome and metabolome were assayed up to 3 years. Greater clinical improvement was seen in RYGB than in DWM for multiple outcomes, including reductions in body weight (assessed by BMI), glycemia (assessed by HbA1c), triglycerides, HDL cholesterol, blood pressure, and reductions in the number of antidiabetic, antihypertensive, and lipid-lowering medications^5^. Moreover, all RYGB participants achieved 10% weight loss before 3 months, whereas only 37% of DWM participants achieved 10% weight loss before 3 months. Given that participants in SLIMM-T2D were randomized to either RYGB or DWM, we can infer causal effects of RYGB (relative to the DWM control) on the outcomes at, for example, one year, and then ask if any clinical outcome or analyte measured at 3 months mediates this causal effect. Identifying mediators among approximately 2000 measured analytes would be a key step to develop new nonsurgical approaches to weight loss and glycemic control in T2D. Many mediation methods are available, but they have been found to lack power in high-throughput studies^22^. A previous comprehensive comparison of mediation methods concluded that the best balance between false positive rate and power was offered by the joint significance method^23^. The joint significance method^23^ in our example assesses if two tests are jointly significant for each analyte: (I) is RYGB associated with analyte abundance at 3 months? and (II) is analyte abundance at 3 months associated with HbA1c at one year given RYGB? The joint significance method was also recommended by another comparative study^22^, where a bootstrap-based method that assesses the product of effects I and II^24^ performed only slightly worse than the joint significance test. However, the bootstrap-based method could not be used in their genome-wide application, since it was too computationally expensive to perform sufficient numbers of bootstraps to reach genome-wide significance^22^. The joint significance method was also shown to control its false-positive rate in theory and to be more powerful than the commonly-used product significance method for mediation^25^. Another popular mediation test not included in these comparative simulations is based on the potential outcomes framework^26^. This framework supports very general causal models, but also assesses significance with the bootstrap or other resampling methods, and so is also computationally burdensome for high-throughput data.

**Fig. 1:**
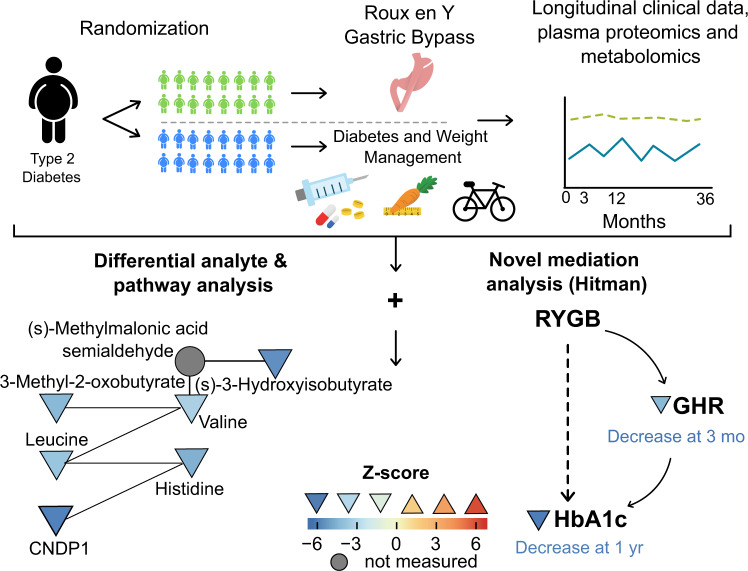
Graphical overview shows study flow including clinical study schematic (top) and analysis (bottom). Differential analyte and pathway analysis (bottom left) identified a network composed of top analytes from Valine, Leucine, and Isoleucine Degradation and Beta-Alanine Metabolism. Mediation analysis (bottom right) identified GHR as mediator of decrease in HbA1c. Triangles denote measured proteins or metabolites, while circles denote proteins or metabolites in the pathway not measured; symbols are colored according to between-group Z scores, using heat map indicated, with the orientation of triangle also indicating up- or downregulation, respectively. Source data are provided as a Source Data file.

Here we report the development of a mediation method termed High-throughput mediation analysis (Hitman) and apply this method to proteomic and metabolomic data from the SLIMM-T2D randomized trial. We identify and validate the growth hormone receptor (GHR) as a mediator of the metabolic impact of RYGB (Fig. 1).

## Results

### Clinical characteristics of participants

Of the 38 SLIMM-T2D participants, proteomic and metabolomic data at baseline, the 3 month time point, and at 1 year were available from 35 participants. Metabolic characteristics of these 35 participants did not differ between groups at baseline (Supplementary Data 1), and HbA1c and BMI of those with proteomics or metabolomics measurements did not differ from the SLIMM-T2D participants within their group at any time point (Supplementary Fig. 1). After 3 years, no DWM participants achieved study-defined glycemic goals (HbA1c < 6.5% and fasting glucose < 126 mg/dL), whereas eight RYGB participants did, and seven of these eight participants were not receiving any anti-diabetes medications^5^. The metabolic characteristics per participant per time point are tabulated in our Zenodo repository, which also includes participants’ diabetes medication use per medication class per time point.

### Longitudinal analysis of the plasma proteome

**Fig. 2:**
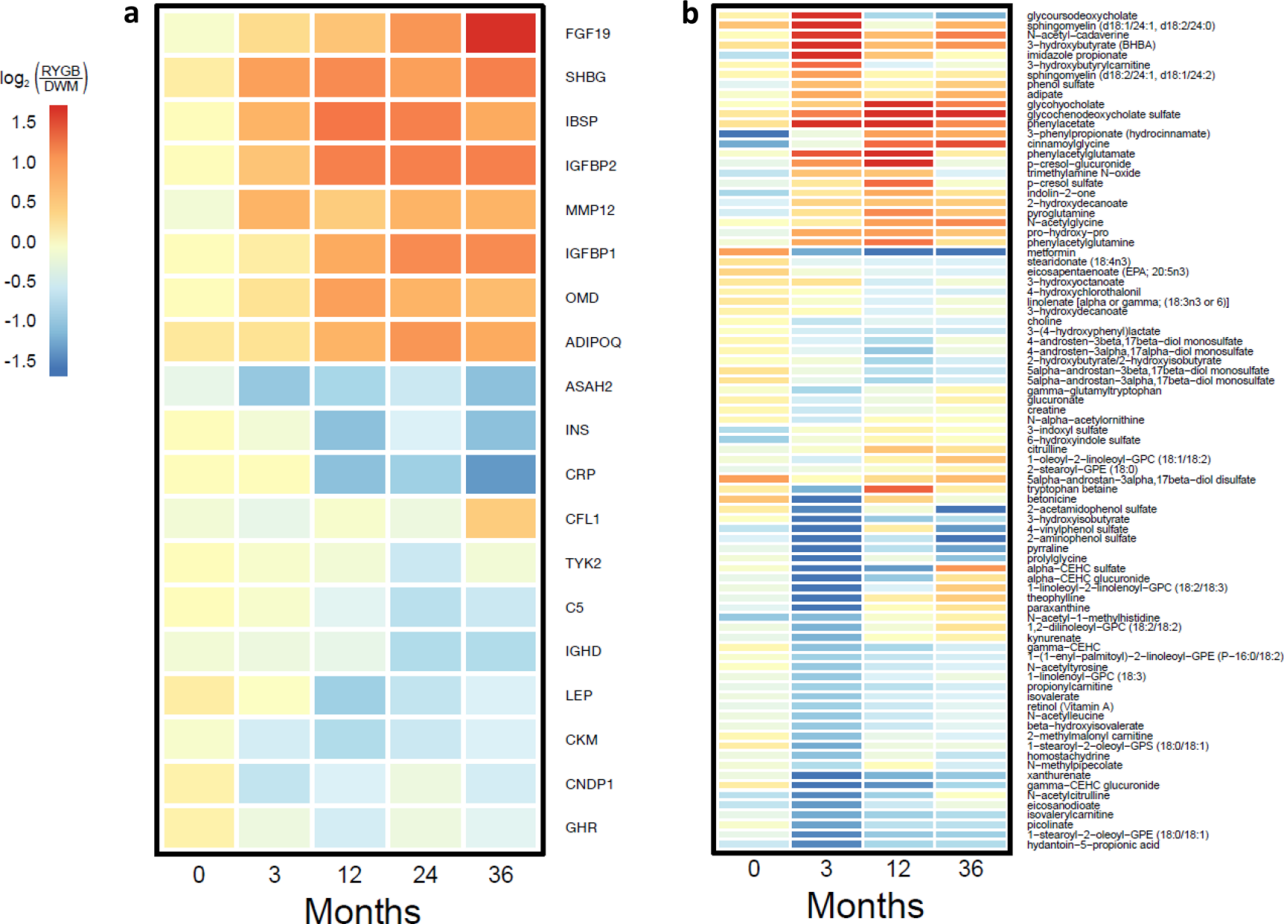
Differentially abundant analytes with time. Heatmaps of **a** proteins and **b** metabolites. Heatmap of log_2_(RYGB/DWM) at all time points (post-baseline log_2_ abundance values are baseline-corrected) for analytes that are differentially abundant at any time point (FDR < 0.15 and fold-change absolute value > 1.5). The range of colors show log_2_(RYGB/DWM) from −1.6 to 1.6, which corresponds to fold changes from −3 to 3. Analytes with a larger absolute value of fold-change are shown as only having fold change absolute value of 3, so that weaker fold changes are easily observed. Source data are provided as a Source Data file.

Fasting proteomics were profiled at baseline, the 3 month time point, and years 1, 2, and 3 in RYGB and DWM. At baseline (pre-randomization), there were no statistically significant proteins (FDR < 0.15). For post-randomization time points, reduction in BMI was greater in RYGB, so groups were compared both with and without adjustment for each person’s BMI change. Differences in the baseline-corrected proteome in RYGB vs. DWM (i.e., differences between groups in changes from baseline) emerged at the 3 month time point, with 14 significant proteins in the unadjusted analysis and 8 in the BMI-adjusted analysis. Differences of baseline-corrected protein abundance in RYGB vs. DWM persisted at years 1, 2, and 3. The baseline-corrected log_2_ fold change values for the 19 proteins differentially abundant with fold-change absolute value >1.5 at any time point without BMI adjustment are depicted in the heatmap in Fig. 2a. Statistics for all comparisons in all analytes are presented in Supplementary Data 2.

We observed several proteins with important roles in systemic metabolism to be significant in unadjusted analyses at several time points, but never significant with BMI adjustment. We considered such analytes as potentially regulated by weight-dependent mechanisms. For example, proteins upregulated post-baseline included sex hormone binding globulin (SHBG), upregulated at all post-baseline time points, and adiponectin, upregulated at 12, 24, and 36 months. Conversely, GHR was downregulated at all post-baseline time points; leptin was downregulated at 12 and 24 months, while C-reactive protein and insulin were downregulated at 12 and 36 months. Multiple complement factor proteins were downregulated at 12, 24, and 36 months, including complement factor H (CFH), C1s, and C5.

Several proteins remained significant after BMI adjustment, indicating potential weight-independence. These include increases in (1) bone structural proteins integrin-binding sialoprotein (IBSP), which was increased at all post-baseline time points in the unadjusted analysis and at 3 and 12 months in the BMI-adjusted analysis, and osteomodulin (OMD), which was increased with and without BMI adjustment at 12, 24, and 36 months, (2) fibroblast growth factor 19 (FGF19), the intestinally-derived target of bile acid and FXR signaling^15^, which was increased with and without BMI adjustment at 36 months, and (3) insulin-like growth factor binding protein IGFBP2, recently shown to be increased after RYGB^27–29^, potentially via improved leptin sensitivity^30^, was increased at all post-baseline time points in the unadjusted analysis and at the 3 month time point in the BMI-adjusted analysis. Of the complement factors, only complement factor B (CFB) remained significant after BMI adjustment, at 12, 24, and 36 months.

Upregulation of IGFBP2 (>50%, FDR < 5% at all post-baseline time points in SOMAscan, Supplementary Fig. 1a) was confirmed by ELISA, which showed significant differences in baseline-corrected values (*p* < 0.05) at 12, 24, and 36 months (Supplementary Fig. 2b), and ELISA changes per person over time were significantly correlated to corresponding SOMAscan changes (*r* = 0.78, *p* < 10^−7^).

### Longitudinal analysis of the plasma metabolome

At baseline (pre-randomization), there were no significant differences in the fasting metabolome between groups. Significant differences in the baseline-corrected metabolome emerged at the 3 month time point, with 96 metabolites differentially abundant in the unadjusted analysis and 74 in the BMI-adjusted analysis; 45 metabolites were common to both analyses.

Differences between arms of baseline-corrected metabolite abundance persisted at years 1, 2, and 3. The baseline-corrected log_2_ fold change values in RYGB vs. DWM for the 85 metabolites differentially abundant with fold-change absolute value >1.5 at any time point without BMI adjustment are depicted in the heatmap in Fig. 2b. Although the color scale is the same as that in Fig. 2a to permit comparison, fold changes were much greater for the metabolome. For example, the BCAA-related metabolite 3−hydroxyisobutyrate was 88% lower in RYGB (i.e., down by >9-fold) with significance in both the unadjusted and BMI-adjusted analysis at the 3 month time point.

Prolylhydroxyproline, a marker of bone collagen degradation, was increased in RYGB at the 3, 12, and 36 month time points, and was also increased with BMI adjustment at the 3 and 12 month time points, again suggesting that postoperative bone metabolic changes are independent of weight loss. The abundance of metformin was reduced with or without BMI adjustment at 12 months, in agreement with reduced medication use in the RYGB group. Interestingly, increases in the conjugated bile acid glycochenodeoxycholate sulfate emerged with time, with significance at the 12 month time point in unadjusted analysis and both the 12 and 36 month time point in the BMI-adjusted analysis.

Having both metabolites and proteins on the same subjects is an advantage of our study, so we tested the correlation of top metabolites’ vs. top proteins’ baseline-corrected changes at 3 months (Supplementary Data 2). Multiple analytes were highly correlated. For example, the metabolite most correlated to GHR was prolylhydroxyproline (*r* = −0.68, *p* < 10^−5^, FDR < 0.002); prolylhydroxyproline was also negatively correlated with CNDP1 (beta-ala-his dipeptidase or carnosine dipeptidase 1; *r* = −0.65, *p* < 10^−4^, FDR < 0.004) and positively correlated with IGFBP2 (*r* = 0.69, *p* < 10^−5^, FDR < 0.002). GHR and CNDP1 were positively correlated to the BCAA-related metabolites beta-hydroxyisovalerate and isovalerylcarnitine, and these were negatively correlated with IGFBP2 (FDR < 0.08 for all these correlations).

### Proteomic and metabolomic integrative pathway analysis

We integrated proteomics and metabolomics for pathway analysis by creating a single, integrated dataset, and testing pathways from the Small Molecule Pathway Database^31^, which has pathways composed of both proteins and metabolites. We identified 50 differentially abundant pathways (FDR < 15%) between groups without BMI adjustment at the 3 month time point.

**Fig. 3:**
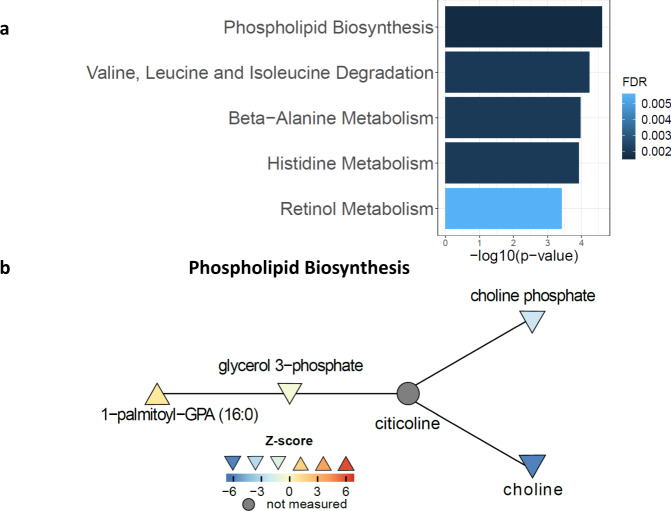
Phospholipid biosynthesis and other top-ranking differential pathways. **a** Top-ranking differential pathways. Graph illustrates –log_10_(*p*-values) and false discovery rates (FDRs), with bars colored by FDR, from Limma Roast’s Mixed statistic^87^ (which is one-sided) adjusted with FDRs. **b** Phospholipid biosynthesis network. Nodes are colored by between-group z-score, whereas unmeasured nodes are colored dark gray. Orientation of triangle also indicates the directionality of regulation. Connections are from the Pathway Commons network. Source data are provided as a Source Data file.

The top-ranking pathways are presented in Fig. 3a. The top-ranking pathway is Phospholipid Biosynthesis (Fig. 3b), whose top analytes were choline (Supplementary Fig. 2c) and choline phosphate (Supplementary Fig. 2d), both reduced in RYGB relative to DWM at the 3 month time point. Choline was also reduced at 12 and 36 months, and the 3 month time point with BMI adjustment, but choline phosphate was not significant in other comparisons.

**Fig. 4:**
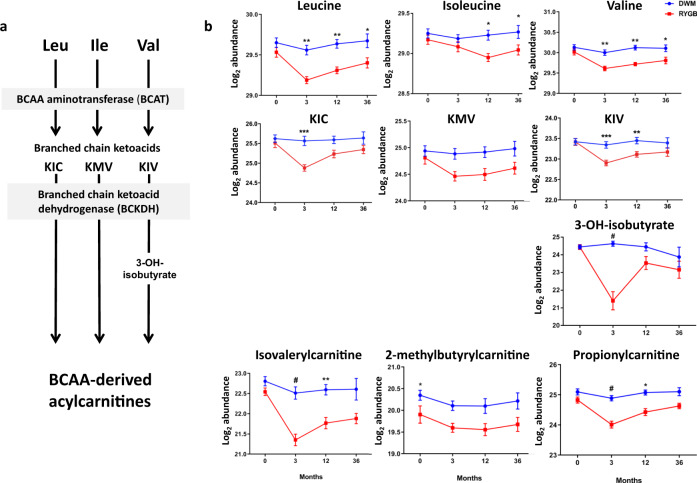
Branched chain amino acids (BCAA) and downstream metabolites. **a** Schematic of BCAA metabolic pathway showing measured analytes. KIC, KMV, and KIV indicate the branched chain ketoacids ketoisocaproate, ketomethylvalerate, and ketoisovalerate, respectively. **b** Relative abundance of measured analytes, aligned to position in pathway in (**a**). Data were analyzed by two-sided moderated t-tests; post-baseline time points were analyzed using change from baseline. Data are reported as mean ± SEM on the log2 scale. Analysis was derived from samples from independent human participants. RYGB at 0, 3, 12, and 36 months: *n* = 19, 18, 19, and 13; DWM: *n* = 19, 18, 16, 9. Nominal *p*-values are as follows: Leucine: 3 mo **= 0.001, 12 mo **= 0.0041, 36 mo *= 0.03; Isoleucine: 12 mo *= 0.017, 36 mo *= 0.036; Valine: 3 mo **= 0.0019, 12 mo **= 0.0013, 36 mo *= 0.016; KIC: 3 mo ***= 0.00016; KIV: 3 mo: ***= 0.00026, 12 mo: **= 0.0045. 3-OH-isobutyrate: 3 mo: #<0.0001; isovalerylcarnitine: 3 mo: #<0.0001, 12 mo: **= 0.002; 2-methylbutyrylcarnitine: 0 mo: *= 0.025; propionylcarnitine: 3 mo: #<0.0001, 12 mo: *= 0.016. isobutyrylcarnitine: 0 mo: *= 0.024. Source data and FDRs are available in Supplementary Data 2.

The second pathway was Valine, Leucine, and Isoleucine Degradation (schematic in Fig. 4a). Its top analytes (Fig. 4b), reduced at the 3 month time point in both the unadjusted and BMI-adjusted analysis, were the BCAAs valine and leucine, the ketoacid 3-methyl-2-oxobutyrate (ketoisovalerate), and the valine catabolic intermediate 3-hydroxyisobutyrate. All but 3-hydroxyisobutyrate were also reduced at 12 months; with BMI adjustment, none were significant at 12 or 36 months, suggesting an important contribution of weight loss during the early postoperative period.

Similar patterns were observed for other ketoacids and downstream acylcarnitines. For example, propionylcarnitine, a C3 acylcarnitine product of valine and isoleucine metabolism, was reduced in RYGB at the 3 month time point in both the unadjusted and BMI-adjusted analyses (but not at later time points with or without BMI adjustment) and was also the top analyte of the related pathway Oxidation of Branched Chain Fatty Acids.

**Fig. 5:**
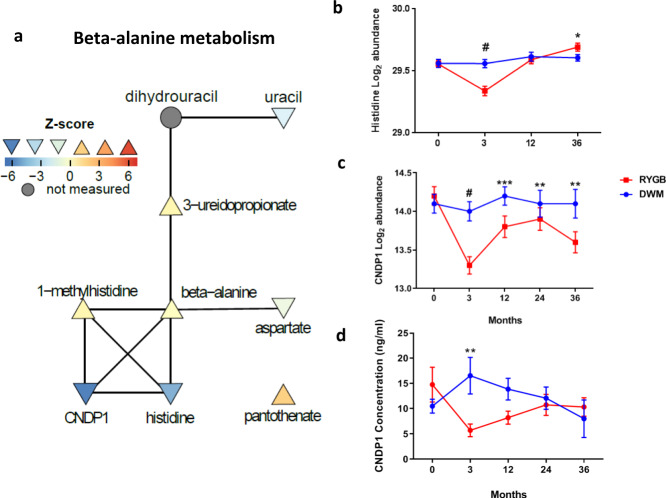
Beta-alanine metabolism. **a** Network nodes are colored by between-group z-score, whereas unmeasured nodes are colored gray. Orientation of triangle also indicates the directionality of regulation. Connections are from the Pathway Commons network. **b** Log_2_ abundance of histidine measured by metabolomics in samples from independent human participants at baseline, 3, 12, and 36 months from 19, 18, 16, and 9 DWM and 19, 18, 19, and 13 RYGB participants. **c** Log_2_ abundance of CNDP1 measured by SOMAscan, measured in samples from independent human participants at baseline, 3, 12, 24, and 36 months from 19, 19, 16, 10, and 9 DWM and 19, 19,19, 15, and 14 RYGB participants. **d** CNDP1 plasma levels measured by ELISA at baseline, 3, 12, 24, and 36 months from 10, 10, 10, 6, and 4 DWM participants and 10, 10, 9, 5, and 6 RYGB participants, respectively. Data were analyzed by two-sided moderated t-tests; post-baseline time points were analyzed using change from baseline. Data in **b**−**d** are reported as mean ± SEM. Nominal *p*-values are as follows: **b** histidine: 3 mo: #<0.0001, 36 mo: *=0.019; **c** CNDP1 (SOMAscan): 3 mo: #<0.0001, 12 mo: ***=0.00014; 24 mo: **=0.0054; 36 mo: **=0.0067; **d** CNDP1 ELISA: 3 mo: **=0.0036. Supplementary Data 2 includes FDRs and source data for (**b**/**c**); source data for **d** is provided as a Source Data file.

Beta-alanine Metabolism’s (Fig. 5a) and Histidine Metabolism’s top analytes were CNDP1 and its enzymatic product histidine. Histidine was reduced after RYGB at the 3 month time point with or without BMI adjustment but not later (Fig. 5b), and its reduction was correlated with that of CNDP1 (*r* = 0.40, *p* = 0.02). CNDP1 was reduced by 43% after RYGB at the 3 month time point and was reduced at 12 months, and at the 3 month time point with BMI adjustment (Fig. 5c). A previous study was unable to validate SOMAscan CNDP1 measurements^32^. We measured CNDP1 with ELISA and our ELISA-determined CNDP1 levels showed little correlation with SOMAscan levels per time point, but changes within person over time showed stronger correlation (*r* = 0.21, *p* = 0.055). We confirmed significant reductions in CNDP1 after RYGB, demonstrating a 68% decrease in baseline-corrected change for RYGB vs. DWM (*p* = 0.004 at the 3 month time point, Fig. 5d).

Retinol Metabolism’s top analyte was retinol (vitamin A), whose baseline-corrected abundance decreased by 33% in RYGB at the 3 month time point, when it was also decreased with BMI adjustment, but over time retinol reverted towards baseline in both groups (Supplementary Fig. 3a). Changes in retinol were significantly correlated to changes in retinol-binding protein 4 (RBP4), shown in Supplementary Fig. 3b, over all time points (*r* = 0.33, *p* = 0.001). Given the role of RBP4 as a mediator of insulin resistance^33^, we measured RBP4 and its partner transthyretin (TTR) in a random subset of 12 subjects by quantitative western blot. From these western blots, RBP4 and TTR were not different between-arms at the 3 month time point (Supplementary Fig. 3c, d), but RBP4 changes (i.e., differences per person over time) correlated significantly to RBP4 changes by SOMAscan (*r* = 0.28, *p* = 0.03), and changes in both RBP4 and TTR significantly correlated to each other (*r* = 0.63, *p* < 10^−5^) and to changes in retinol (both *r* > 0.6, *p* < 10^−4^).

### Development of high-throughput mediation analysis (Hitman)

**Fig. 6:**
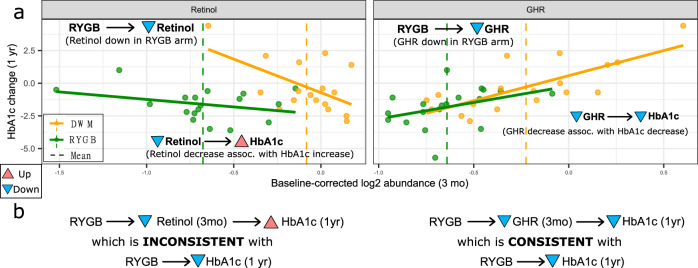
GHR is a consistent mediator of change in HbA1c. Retinol is an inconsistent mediator (i.e., it suppresses or inhibits the causal effect; left side) whereas GHR is a consistent mediator of HbA1c change at 1 year (right side). **a** *X*-axis represents log_2_ abundance change from baseline to the 3 month time point, and *Y*-axis represents HbA1c change from baseline at 1 year. **b** Shows putatively causal links with arrows based on (A) and direction of change with triangles pointed up (increase) or down (decrease). Source data are provided as a Source Data file.

We sought to identify analytes whose change in abundance at the postoperative 3 month time point mediated RYGB’s improvement in diabetes control at one year. We know that RYGB improves diabetes outcomes from prior studies, so we can be confident that RYGB actually improves diabetes, as we observe, rather than the opposite direction of effect (in which case RYGB would exacerbate diabetes). A reasonable mediation method to use would be the joint significance method. However, one drawback of the joint significance test and other mediation methods when the direction of the causal effect of the exposure on the outcome is known is that they do not account for the direction of effect of the mediator, so they could call as significant an “inconsistent” mediator, which suppresses or inhibits the causal effect (Fig. 6)^34^. For example, relative to DWM, RYGB decreases the baseline-corrected abundance of retinol at the 3 month time point, and RYGB decreases baseline-corrected 1 year HbA1c (Fig. 6, left side). However, a decrease in retinol is associated with an increase in HbA1c—a direction of mediation inconsistent with RYGB decreasing HbA1c, so retinol does not appear to mediate RYGB’s beneficial effect.

A second drawback of the joint significance and other mediation methods is that they are intended for testing only one or a few mediators. Thus they do not employ powerful statistical methods for high-throughput data, such as linear regression modeling with empirical Bayesian modeling of an analyte’s variance with the R package Limma^35^, which has been validated in multiple omics platforms, and is particularly powerful for small sample sizes.

To address these limitations, we developed high-throughput mediation analysis (Hitman). Hitman is designed for studies where the exposure causes a significant change in the outcome in the same direction as known a priori and high-throughput mediators are assayed. Hitman improves power by accounting for the direction of effect and by employing empirical Bayesian linear modeling^35^. If a mediator’s direction of effect is consistent with the prespecified direction of the causal effect of the exposure on the outcome, its *p*-value from the joint significance test is halved. Otherwise, its *p*-value becomes one. In the case of the inconsistent mediator retinol, Hitman assigns a *p*-value of one. The description of the algorithm, implemented in our R package *Hitman*, mathematical proof that Hitman provides valid p-values when there is a causal effect whose direction is known, and details of our simulations and their results are provided in Supplementary Note 1.

Using simulations similar to those of Barfield et al.^36^ and MacKinnon et al.^23^, we compared Hitman against the joint significance test and the potential outcome mediation test^26^, which had not been included in previous simulations. Consistent with our mathematical proof, our simulation using 50 samples demonstrates that Hitman properly controls its false positive rate when the causal effect’s direction is known and specified prior to the analysis. Our simulations also test the ability of the methods to detect consistent mediators, and confirm that Hitman has more power. For example, when the true causal mediation effects are of a size termed “small”^23^, the joint significance and the potential outcome mediation test identify mediation in <2.5% of simulations, whereas Hitman identifies mediators in >6%, which is significantly greater than both (odds ratio (OR) = 2.89, 95% CI = [2.48, 3.39], *p* < 10^−15^). When the true effect is of “medium” size^23^, the proportion of simulations that identified mediation by the joint significance test and the potential outcome mediation test were 53.9 and 57.4%, respectively, whereas Hitman identifies 68.5% (OR = 1.62, 95% CI = [1.52, 1.71], *p* < 10^−15^).

The directional nature of Hitman accounts for much but not all of Hitman’s power, since the empirical Bayesian variance estimation adds power, particularly with smaller sample sizes. Smaller sample sizes are common in basic research with model organisms, where variance between units in a group is expected to be small. Using Limma^35^ also allows for testing high-throughput data types that are not normally distributed, such as RNA-seq^37^. We demonstrate the benefit of using Limma by simulating as above but with only 15 samples. We also compare these simulations against a simplified version of Hitman that does not use Limma, which is applicable to scenarios other than high-throughput data. We find with medium effect size that the joint significance and the potential outcome method have power <7%, the simplified version of Hitman that does not use Limma has power 11.8%, and Hitman has the power of 16.1%, which is significantly greater than the others (OR = 1.43, 95% CI = [1.32, 1.55], *p* < 10^−15^).

We next conducted omics simulations using datasets of 500 analytes where *p*-values were adjusted with the false discovery rate (FDR). We simulated sample sizes of 15 and 50, either 1, 5, or 25 consistent mediators, and 200 analytes associated with the exposure but not the outcome in three scenarios: (1) analytes are independent and mediators are consistent; (2) analytes are dependent on each other and mediators are consistent; and (3) analytes are dependent on each other and there are an equal number of consistent and inconsistent mediators of equal effect. We compared the joint significance method, Hitman, and the version of Hitman that does not use Limma. We did not include the potential outcome mediation test in these simulations because of its computational cost. We modeled dependence between analytes based on the dependence structure from the GTEx expression dataset^38^, which was adjusted for measured and inferred^39^ covariates, like the processing by Oliva et al.^40^.

We found that power increased with more samples and fewer mediators, whether these mediators were consistent or inconsistent, and that including dependence from GTEx had little effect on the results, most likely because its covariates were properly accounted for. We also found that Hitman controls its FDR in all scenarios and has the most power, with the greatest advantage when *N* = 15. For example, in scenario 1 when *N* = 15 and there was one true mediator, the joint significance method had power of 31.5%, whereas Hitman had power of 42.4% (OR = 1.6, 95% CI = [1.33, 1.93], *p* = 5*10^−7^).

### Mediation analysis: analytes, clinical data, and pathways

We applied Hitman to identify analytes whose early change (baseline to 3 month time point) mediates HbA1c improvement at one year (Supplementary Data 2). The only significant analyte was growth hormone receptor (GHR; *p* = 10^−4^, FDR = 0.12; Fig. 6, right), which was reduced by 24% in RYGB at the 3 month time point. We also tested the change in proteins and metabolites at the 3 month time point vs HbA1c change at the 18, 24, and 36 month time points. However, we found no significant protein or metabolite mediators of HbA1c at these later time points, potentially because after 12 months the number of participants with HbA1c measurements declines substantially, or there are additional secondary changes, such as progressive weight loss or inter-individual variation in diet or other factors (Supplementary Data 1).

As a comparison to Hitman, we applied the joint significance method to test mediation of proteins and metabolites whose change at the 3 month time point mediates HbA1c improvement at one year. We found similar top analytes, but with weaker significance, consistent with our simulations demonstrating that Hitman offers significantly more power (Supplementary Note 1). The top-ranking analytes identified by the joint significance method were GHR (*p* = 2.3*10^−4^; FDR = 0.26) and prolylhydroxyproline (*p* = 10^−3^; FDR = 0.73). Prolylhydroxyproline was also the second-ranking analyte in Hitman (*p* = 5*10^−4^, FDR = 0.32; Supplementary Data 2).

We next asked whether the early postoperative change (baseline to the 3 month time point) in 40 clinical markers mediated HbA1c improvement at one year. The top-ranking early postoperative mediators identified by Hitman were 6 min walk test distance and BMI (*p* < 0.03, FDR = 0.4; Supplementary Data 2). Strikingly, GHR was more significant (by raw and FDR-adjusted *p*-value) than any clinical markers, suggesting its utility as a potential clinical biomarker.

We next sought to test the mediation of our integrated pathways. Several approaches test pathway mediation^41,42^. We utilized the Camera procedure to test pathway enrichment as it can utilize Hitman’s scores and accounts for the correlation between genes^43^. We first tested for pathways whose change at the 3 month time point mediate HbA1c improvement at 1 year, but none were significant.

Given that improvements in glycemic control after RYGB are known to be related to changes in insulin sensitivity and/or insulin secretion^44^, we applied Hitman to identify analytes whose change from baseline at the 3 month time point mediated insulin secretion and insulin sensitivity change from baseline at 1 year (Supplementary Data 2) and followed this with integrative pathway mediation analysis. Insulin secretion, defined as the change in insulin from 0 to 30 min during a mixed meal tolerance test, was improved in RYGB vs. DWM, as expected (means of changes: RYGB = 37.4, DWM = 0.808; *p* = 0.001). No single analyte was identified as a significant mediator, but one pathway, Caffeine Metabolism, significantly mediated improved insulin secretion (FDR < 10^−4^). Similarly, insulin sensitivity, defined by reduction in HOMA-IR, also improved in RYGB vs. DWM at 1 year (means of changes: RYGB = −2.12, DWM = −0.102; *p* = 0.02). No individual proteins or metabolites were significant mediators of insulin sensitivity, but many top-ranking analytes were BCAA-related metabolites. Consequently, pathway analysis identified Valine, Leucine, and Isoleucine Degradation as a significant pathway mediator of insulin sensitivity (FDR < 10^−7^), together with 12 other pathways, primarily involved in lipid and amino acid metabolism (Supplementary Data 2).

### Validation of growth hormone receptor as candidate mediator

GHR was decreased by 24% at the 3 month time point after RYGB, relative to DWM, with similar reductions of 35, 28, and 30% at 12, 24, and 36 months, respectively. Robust GHR mediation led us to hypothesize that reductions in plasma GHR reflected reduced tissue content or altered receptor shedding, and thus could be associated with reduced growth hormone (GH) signaling. In the liver, GH is best recognized as a determinant of insulin-like growth factor 1 (IGF1) secretion during periods of nutritional sufficiency, but GHR also exerts complex pro-diabetogenic effects on glucose metabolism via IGF1-independent pathways^45^. Indeed, IGF1 was not identified as a mediator by Hitman, and neither SOMAscan nor ELISA measures of IGF1 differed between groups at the 3 month time point (Supplementary Fig. 4). Levels of IGFBP1 and IGFBP2, which are repressed by GH signaling, were increased in RYGB vs. DWM at multiple time points (Fig. 2a and Supplementary Fig. 2). Plasma GH levels did not differ at the 3 month time point, but were >6-fold higher in baseline-corrected RYGB vs. DWM at 12 months (Supplementary Fig. 4). Collectively, these data support the hypothesis that pathway-selective growth hormone resistance in post-RYGB participants could contribute to sustained improvements in glucose metabolism^46^.

**Fig. 7:**
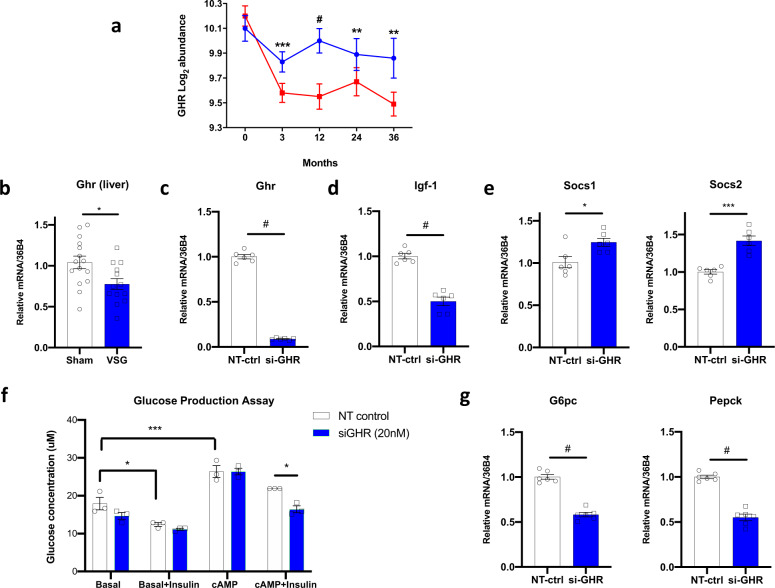
GHR is reduced after bariatric surgery in humans and rodents and regulates hepatic glucose metabolism. **a** GHR in plasma is reduced after RYGB in humans, measured by SOMAscan in samples from independent human participants at baseline, 3, 12, 24, and 36 months from 19, 19, 16, 10, and 9 DWM and 19, 19,19, 15, and 14 RYGB participants. **b** *Ghr* expression in liver, assessed by qRT-PCR, from diet-induced obese rats, 8 weeks post-VSG bariatric surgery (VSG) or sham controls (Sham) (*n* = 13 VSG, *n* = 15 sham). **c**–**g** Expression of GH signaling/axis genes, assessed by qRT-PCR, in cells treated with siRNA targeting Ghr (si-Ghr) or non-targeting scrambled control siRNA (NT-ctrl) (*n* = 6 per group). **c** Ghr, **d** Igf-1, and **e** Socs1/2 in mouse primary hepatocytes treated with siRNA-Ghr or NT-control. **f** Glucose production in response to insulin, cAMP, and insulin/cAMP after si-Ghr or NT-ctrl. **g** Expression of gluconeogenic genes G6pc and Pepck in response to si-Ghr or NT-ctrl. Data are reported as mean ± SEM. For **a** the *p*-values are nominal, using a two-sided T-test; FDRs are reported in Supplementary Data 2. For **b**–**e** and **g** two-sided t-tests were applied; for **f** two-way ANOVA with Bonferroni’s multiple comparison test was applied in GraphPad Prism. *P* values are as follows: **a**: GHR: 3 mo: ***= 0.00099; 12 mo: #<0.0001, 24 mo: **= 0.0024, 36 mo: **= 0.0013. **b**: *= 0.0155, **c**: # <0.0001, **d**: # <0.0001; **e**: Socs1: *= 0.017, Socs2: ***= 0.0002; **f**: basal vs. insulin in NT control: *= 0.0322; basal vs. cAMP in NT control: ***= 0.0004; NT control vs. siGHR in cAMP + insulin: *= 0.0328. **g**: G6pc and Pepck both #<0.0001. Source data for **a** is provided in Supplementary Data 2. Source data for panels **b**–**g** are provided in the Source Data file. For expression analysis, two independent experiments were performed, while glucose production data represent a single experiment.

Given that reduction in plasma GHR was a top-ranking mediator of improvements in glycemia, we asked whether expression of *Ghr* in the liver (where *Ghr* is most highly expressed) is similarly reduced after experimental bariatric surgery in rodents. We analyzed *Ghr* expression in liver of HFD-fed rats 8 weeks after vertical sleeve gastrectomy. Expression of *Ghr* was reduced by 26% (*p* < 0.05) in VSG-treated as compared with sham rats (Fig. 7b). Thus, reductions in hepatic *Ghr* follow both VSG and RYGB^47^ forms of bariatric surgery in rodents, paralleling the reduction in plasma GHR we observed in humans following RYGB.

Given that changes in hepatic transcriptional patterns could reflect both the direct impact of surgery and secondary effects of altered metabolism, we next investigated whether experimental reduction of *Ghr* could affect glucose metabolism in a cell-autonomous fashion in mouse primary hepatocytes. siRNA-mediated *Ghr* knockdown led to a > 90% reduction in expression of *Ghr* and 50% reduction in expression of *Igf-1* (Fig. 7c, d) as well as increased expression of the GH signaling inhibitors *Socs1* and *Socs2* (Fig. 7e). Furthermore, *Ghr* knockdown enhanced insulin action to repress gluconeogenesis, as revealed by (1) increased insulin-mediated suppression of cAMP-stimulated glucose production (Fig. 7f), and (2) downregulation of the gluconeogenic enzymes *G6pc* and *Pepck* (Fig. 7g). Therefore, inhibition of hepatocyte GH signaling reduces transcriptional activation of gluconeogenesis and reduces glucose production. Since these key pathways contribute to hyperglycemia in T2D, reductions in GH signaling could contribute to improved glucose metabolism.

## Discussion

RYGB yielded weight loss and improvement in multiple clinical parameters. Our proteomic and metabolomics analysis in the fasting state revealed both weight-dependent and -independent protein and metabolite changes. Weight-dependent changes included decreases in insulin, leptin, proinflammatory proteins, amino acids, and lipid-related metabolites; these changes are consistent with improved insulin sensitivity, loss of adipose mass, and reductions in inflammation observed in prior studies of RYGB^9,10,48,49^. In contrast, the differential abundance of several analytes linked to bone or bile acid metabolism remained after BMI adjustment, suggesting weight-independent mechanisms underlying these changes. For example, the highly abundant bile acid and FXR ligand glycochenodeoxycholate sulfate and the intestinal FXR-dependent hormone FGF-19 were upregulated at later time points even with BMI adjustment in participants randomized to RYGB, consistent with prior reports in patients post-RYGB^11,12,14,15^ and in those with hypoglycemia after gastric bypass^50^.

Valine, Leucine, and Isoleucine Degradation was the most significantly changed pathway at the 3 month time point between groups and a significant HOMA-IR pathway mediator. There were robust post-RYGB decreases in BCAA and multiple downstream catabolic intermediates, including C3 and C5 acylcarnitines. Our results are in agreement with reduced BCAA in response to RYGB in nonrandomized studies^9,10,51,52^ and prior findings of increased BCAA in insulin resistance^53,54^. Lower BCAA levels post-RYGB may be a consequence of weight loss-related improvements in insulin sensitivity or altered microbial metabolism^18^, but may also contribute directly to improved insulin sensitivity^55^. Our data reveal a marked 88% reduction in the valine catabolic intermediate 3-hydroxyisobutyrate at the 3 month time point, which is particularly interesting since 3-hydroxyisobutyrate can exit mitochondria and serve as a signaling molecule, promoting muscle lipid uptake and insulin resistance^56,57^.

The significant protein putative mediator of improved glycemic control was GHR, which was reduced after RYGB. GHR at the 3 month time point is a more significant mediator of glycemia at 1 year than any clinical markers, including BMI, indicating that GHR may have clinical significance as a biomarker of bariatric surgery or as a therapeutic target. In agreement, liver *Ghr* expression was significantly reduced after VSG in rats with dietary obesity. These data are consistent with the >50% reduction in *Ghr* mRNA expression and protein (assessed by PCR and immunohistochemistry) in liver and multiple intestinal segments recently reported at both 9 days and 9 weeks post-RYGB in mice^47^. These reductions in *Ghr* were also observed in weight-matched mice, indicating weight-independence. In our human cohort, reduction in GHR was closely correlated with reductions in BCAA-related metabolites, which have also been linked to improved post-surgical metabolism.

Beyond reductions in GHR content, we observed changes in plasma levels of downstream targets of GH signaling, with a robust increase in IGFBP1 at the 3 month time point and both IGFBP1 and IGFBP2 at 12, 24, and 36 months; these changes are concordant with reduced GH effect within IGF1-independent pathways regulating hepatic and systemic metabolism. The time course of these effects provides potential clues. Reduction in GHR at the 3 month time point was accompanied by an increase in the GHR-dependent target IGFBP1^47^. By contrast, increases in GH measured by ELISA were not observed at the 3 month time point, but only emerged at 12 months. Taken together, these data support the hypothesis that late postoperative increases in GH may reflect both acquired GH resistance following RYGB, together with the reversal of obesity- and inflammation-associated reductions in GH secretion^58^. The emergence of increases in GH at 12 months in the surgical group could also contribute to sustained lipolysis in adipose tissue^59^. Additional experiments will be required to identify the tissue-specific changes in GH action associated with improved metabolic control after RYGB in patients with T2D.

Given that GH excess (due to acromegaly or pharmacologic use of GH in adults) is associated with increased glucose levels and diabetes in humans^60^, a net reduction of GH signaling would be expected to improve systemic metabolism. Indeed, rodent models with experimental ablation of either *Gh* or *Ghr* have increased healthspan, delayed aging, and improved insulin sensitivity^45,61^. Mice with liver-specific ablation of *Ghr* have impaired gluconeogenesis, with overt hypoglycemia^62^. Moreover, inhibition of GH action by the GHR antagonist pegvisomant improves systemic insulin action in humans^46^.

Our data in isolated primary hepatocytes with experimental knockdown of *Ghr* provide further support for an important role for reduction in GHR-dependent signals as a mediator of improved glucose metabolism. Efficient *Ghr* knockdown reduced expression of gluconeogenic enzymes *Pepck* and *G6pc*, and enhanced the action of insulin to repress glucose production. Taken together, our study adds to the growing evidence that RYGB decreases GHR-dependent signaling in the liver, and supports that this reduction may contribute to lowering of glucose and robust improvements in diabetes control^63–66^.

We have developed and applied a mediation method that can be applied to studies where samples are randomized to a treatment (or can be modeled as randomized given covariates) whose causal effect on the outcome is consistent with prior knowledge and these samples’ potential mediators are assayed. As others have noted^67^, mediation analyses can be misled by confounding variables, such as an unmeasured factor that both affects GHR at the 3 month time point and (independently of GHR) affects HbA1c at one year. To avoid confounding among measured mediators, some high-throughput mediation approaches decompose measured analytes to identify latent mediators that are independent of measured confounders^42,68^. However, these latent mediators are composed of multiple analytes and can be difficult to interpret and follow-up experimentally. Furthermore, in complex biological systems, many potential confounders are unmeasured, such as microbiome changes in this study, so confounders’ impact often cannot be prevented by decomposing measured analytes. To avoid confounding, it is helpful to control for relevant covariates. For example, in this study Hitman analyzed changes within individuals, which obviates the need to control for some demographic variables. For our omics simulations, we measured inter-gene dependence in GTEx after controlling for previously identified covariates and unknown factors using surrogate variable analysis (SVA)^39^, which decreased dependence. However, Hitman tests mediators individually and does not attempt to control for confounders or inter-analyte dependence, so its results should be considered exploratory, like other causal mediation methods that test analytes individually in omics datasets^41^. Future work should focus on combining Hitman with methods that consider multiple mediators simultaneously, such as pathway analyses^41^, and account for some level of confounding^69^.

As required for a directional (“one-sided”) test, the direction to be tested in Hitman is defined using prior knowledge before Hitman is applied^70^; this must be the same as the observed direction of the exposure’s effect on the outcome. The question of when directional tests are appropriate is often debated, but the primary criteria are that the direction of the test should align with prior evidence^71^ and the scientific hypothesis^72–75^. These two criteria are related since scientific hypotheses are based on prior evidence. For example, multiple studies (including ours) have found that RYGB causes a robust decrease in HbA1c. This provides confidence that RYGB truly decreases HbA1c, which supports the hypothesis that there exist mediators of RYGB’s decrease in HbA1c. If the observed causal effect is not in the pre-specified direction or no information about the direction of a potential causal effect is available, then Hitman should not be applied, as the approach of using a directional test is invalid, and the false positive rate could be inflated by two-fold.

Here we are interested in consistent mediators, which can explain the causal effect of the exposure on the mediator and the mechanism of action^76^. The mediation hypothesis in the inconsistent direction is that there exist mediators that suppress or inhibit RYGB’s reduction of HbA1c. Detecting inconsistent mediators could be of value, as these mediators could be blocked to enhance RYGB’s reduction of HbA1c. If these exist, though, their effect size should be small relative to the magnitude of RYGB-related reduction of HbA1c. If only inconsistent mediators are of interest, though, Hitman can be slightly modified so that consistent mediators are assigned a *p*-value of one and the *p*-value of inconsistent mediators are halved. Without this modification, Hitman has no power to detect inconsistent mediators.

We acknowledge that profiling semi-quantitative plasma metabolomics and proteomics, with emphasis on the secreted proteome, cannot fully define the pleiotropic effects of bariatric surgery, including both weight-dependent and weight-independent changes in complex inter-organ communication, gut microbiome effects, tissue-specific protein abundance, or flux in metabolic pathways. Moreover, medication usage, activity, and diet composition could impact both clinical and omics differences between groups in the human cohort. Although studies in rodents cannot fully recapitulate human systemic metabolism, we were able to identify and validate changes in *Ghr* and related pathway genes under controlled experimental conditions. The analytes we have identified and validated can be modulated in future studies to determine whether they can be utilized for non-surgical control of glucose metabolism in T2D, and our mediation method can be applied in translational omics studies.

## Methods

### Clinical study

All relevant ethical regulations for work with human participants were followed, and informed consent was obtained from all participants. The protocol was approved by the Partners Healthcare Institutional Review Board, and an independent data monitoring committee reviewed patient safety.

We performed a retrospective, exploratory analysis of fasting plasma samples from the Surgery or Lifestyle with Intensive Medical Management in the Treatment of Type 2 Diabetes (SLIMM-T2D) clinical trial (clinicaltrials.gov:NCT01073020), in which 38 individuals with T2D and obesity were randomized to RYGB using standard operative protocols (*n* = 19) or nonsurgical intensive diabetes weight management (DWM; *n* = 19) and followed longitudinally for 3 years; pre-specified primary study outcomes were previously reported^5^. Metabolic assessments were performed at baseline (pre-randomization) and repeated at the 3 month time point (defined as achieving 10% of initial body weight loss or at 3 months, permitting assessment at similar weight loss in both groups) and at 12, 18, 24, and 36 months.

Blood samples were obtained after an overnight fast and analyzed for HbA1c, plasma glucose, lipids (Quest Diagnostics), and insulin (Mercodia ELISA); additional aliquots were stored at −80 °C and used for the current analysis. Samples with HbA1c were available from 19, 19, 19, 17, 16, and 15 participants from the RYGB arm, and 19, 19, 18, 11, 10, and 10 participants from the DWM arm at the baseline, 3, 12, 18, 24, and 36 month time points. Groups were metabolically similar at baseline^5^.

Meal tolerance and 6 min walk testing were performed at baseline and at 3 and 12 months as described^77^. In brief, Ensure (9 g protein, 40 g carbohydrate, 6 g fat) was consumed over 5 min, and blood was sampled at 30, 60, and 120 min. Insulin sensitivity was calculated by HOMA-IR, while insulin secretion was calculated as the change in insulin from 0 to 30 min during the meal test.

### Proteomic profiling and validation

Plasma proteome profiling was performed using the high-throughput DNA aptamer-based SOMAscan assay platform (SomaLogic, Inc.)^78^. The abundance of 1129 proteins (enriched for extracellular proteins) was quantified as relative fluorescent units (RFU), normalized, calibrated, and log_2_-transformed. Samples were available at baseline, 3, 12, 24, and 36 month time points for 19, 19, 19, 15, and 14 participants in the RYGB arm, and for 19, 19, 16, 10, and 9 participants in the DWM arm (Supplementary Data 1).

Selected proteomic data were validated by ELISA in a subset of fasting plasma samples, including IGFBP2 (22-BP2HU-E01, ALPCO, NH), CNDP1 (F34010, LifeSpan Biosciences, WA), growth hormone (DGH00, R&D Systems, MN), and total IGF-1 (DG100, R&D Systems, MN). RBP4 and TTR were assayed by quantitative western blotting using polyclonal anti-human RBP4 (Dako) and anti-human TTR (Dako) with standard curves of purified human RBP4 or TTR (Sigma) on each blot^79^. Changes per individual over time were tested for positive correlation to corresponding SOMAscan changes with a one-sided test of Pearson correlation.

### Metabolomic profiling

Plasma metabolomics were profiled using a commercial semi-quantitative mass spectrometry-based platform (Metabolon, Inc.) as described^80,81^. In brief, samples were prepared using the automated MicroLab STAR^®^ system from Hamilton Company. Several recovery standards were added prior to extraction for QC purposes. To remove protein, dissociate small molecules bound to protein or trapped in the precipitated protein matrix, and to recover chemically diverse metabolites, proteins were precipitated with methanol under vigorous shaking for 2 min (Glen Mills GenoGrinder 2000) followed by centrifugation. The organic solvent was removed using the TurboVap^®^ (Zymark); after overnight storage under nitrogen, samples were reconstituted in solvents prior to separation using ultrahigh performance liquid chromatography-tandem mass spectroscopy (UPLC-MS/MS) with scan range 70–1000 m/z. Each reconstitution solvent contained a series of standards at fixed concentrations to ensure injection and chromatographic consistency. Raw data were extracted, and peaks were quantified by area-under-the-curve.

The majority of metabolites were identified by confirming a structure with a minimum of two orthogonal properties (here, an accurate mass m/z, accurate mass fragmentation, and a retention time) from a pure reference standard acquired under identical analytical conditions. These metabolites have confidence level 1, as per the Metabolomics Standard Initiative^82^. Those metabolites not officially confirmed based on a standard have confidence level 3 and their biochemical name is marked with an asterisk (*) in the metabolite annotation (Supplementary Data 2 and 3); these are primarily lipids.

Quality control was ensured by assessment of instrument and process variability, determined by calculating the median relative standard deviation (RSD) for the (a) internal standards added to each sample prior to injection into the mass spectrometers, and (b) endogenous metabolites present in 100% of technical replicates of a large pool of extensively characterized human plasma. Values for median RSD for instrument and process variability were 5 and 9%, respectively.

This dataset had 17% of values missing, and the metabolite average abundance from non-missing values was strongly negatively correlated with the metabolite’s proportion of missing values (Spearman rho = −0.59, *p* < 10^−15^). This indicated that missingness is abundance-dependent, i.e., data were missing not at random (MNAR)^83^. For MNAR data, potential imputation approaches include imputing half of the minimum abundance per metabolite (HM) and quantile regression imputation of left-censored data (QRILC), which imputes the left-censored data by randomly drawing values from a truncated normal distribution^84^.

Metabolites that had missing values in more than 85% of samples were filtered out, leaving the dataset with 10% missing values. Missing values were imputed with HM, and abundance values were log_2_-transformed. Samples were available at baseline, 3, 12, and 36 months from 19, 18, 19, and 13 participants in the RYGB arm and from 19, 18, 16, and 9 participants in the DWM arm. Metabolomics were not profiled at 24 months due to cost. Metabolomics data are provided in Supplementary Data 3.

### Differential abundance of proteomics, metabolomics, and pathways

To test differential abundance of log_2_ normalized analytes between groups at baseline, we applied two-sided moderated t-tests with the R package *Limma*^35,85^, and accounted for multiple hypothesis testing by controlling the FDR with the Benjamini−Hochberg method^86^. We analyzed proteins and metabolites separately. Limma applies linear regression modeling with empirical Bayesian methods to improve each analyte’s variance estimation using analytes’ shared systematic variance. At post-baseline time points, we calculated change in analyte abundance from baseline for each individual, and then applied moderated t-tests to test if these changes varied by group. We repeated these post-baseline analyses accounting for each person’s BMI change as a covariate.

Significance for this and other analyses of high-throughput data (either from SOMAscan or Metabolon) was defined as FDR < 0.15. However, significance for analytes measured without high-throughput platforms, such as clinical data (e.g., BMI) or proteins analyzed by ELISA, was defined as *p* < 0.05.

To test the correlation of top metabolites to top proteins, we tested the correlation of baseline-corrected changes, and chose top analytes as those shown in the heatmaps in Fig. 2a, b.

To identify differentially abundant pathways, we constructed an integrated dataset of proteins and metabolites for samples that had both data types, where we averaged SomaLogic probes that had identical gene symbols. We applied Limma’s Roast method to this dataset against Small Molecule Pathway Database^31^ pathways. We were interested in pathways that change, even if, for example, proteins tend to increase and metabolites tend to decrease, so we reported statistics from Roast’s “Mixed” test, which tests if a pathway’s analytes tend to change without regard to direction (i.e., if analyte changes have large absolute values), so a pathway can be significant even if some analytes go up and others go equally down^87^.

To plot top nodes in a network, we plot the most significant analytes as nodes with edges between them from the Pathway Commons^88^ network version 9. When disconnected nodes can be easily connected, we add less significant or unmeasured intermediates. Nodes are colored by their t-statistics, which had sufficiently high degrees of freedom to approach z-scores, and were labeled as such.

In plots of individual analytes with standard error of the mean (SEM), ordinary (i.e., unmoderated) SEM are displayed.

### Mediation analysis

We tested mediation of each clinical variable with the causal chain: group → clinical variable change → clinical outcome change. We tested each clinical variable’s mediation by defining *group* as a binary variable representing RYGB or DWM per individual; *clinical variable change* as each clinical variable’s change between baseline and the 3 month time point per individual; and *clinical outcome change* as the change in clinical outcome between baseline and 12 months per individual.

We tested mediation of analytes with the causal chain: group → analyte change → clinical outcome change, with *analyte change* as each analyte’s change (on the log_2_ scale) between baseline and the 3 month time point per individual and the *clinical outcome change* as the change between baseline and 12 months (or in some cases later time points). As a comparison to Hitman, we similarly tested the mediation of analytes using the joint significance method and using the counterfactual approach with the mediation package^26^ in the R software. In our simulations to evaluate methods, the number of significant simulations was compared statistically with a two-sided Fisher exact test in the R software. We tested these Hitman results against pathways from the Small Molecule Pathway Database (SMPDB)^31^ with the CAMERA pre-ranked pathway analysis method^43^ from the Limma package.

### Validation studies in rodent model of bariatric surgery and in primary mouse hepatocytes

The study complied with all relevant ethical regulations for animal testing and research. All protocols were reviewed and approved by the University of Michigan (Ann Arbor, MI) and Joslin Diabetes Center Animal Care and Use Committees. Rat bariatric surgery was performed in high fat diet (HFD) fed male mice as described^89^. Liver tissue was dissected 8 weeks postoperatively and flash-frozen for subsequent RNA extraction.

### Primary hepatocyte isolation and siRNA-mediated gene knockdown

Primary hepatocytes were isolated from wild-type C57Bl6 male mice at age 8 weeks. After anesthesia (pentobarbital, 50 mg/kg), the vena cava was cannulated (30G) and the portal vein was cut for drainage. The liver was perfused for 5 min with 20 mL of perfusion solution (NaCl 140 mM, Tricine 25 mM, KCl 5.5 mM, EGTA 0.5 mM, KH_2_PO_4_ 0.5 mM, Na_2_HPO_4_ 0.3 mM, pH 7.4) and then for 5 min with 25 mL of digestion buffer (Eagle’s balanced salts (NaCl 115 mM, NaHCO_3_ 25 mM, D-Glucose 5.5 mM, KCl 5.5 mM, CaCl_2_ 0.3 mM, Na_2_HPO_4_ 0.8 mM, MgSO_4_ 0.8 mM), 1% penicillin/streptomycin and collagenase type II) for 5 min. The liver was harvested and minced; the cell suspension was filtered through a 100 μm cell strainer; cells were collected via centrifugation (200 × *g*, 3 min), and the pellet was resuspended in 15 ml of complete DMEM (glucose 450 mg/dL) containing 10% fetal bovine serum (FBS) and 1% penicillin/streptomycin. The cell suspension was mixed with 10 ml of Percoll and centrifuged at 340 × *g*, 10 min); the pellet was resuspended in 25 mL complete DMEM, centrifuged again (200 × *g*, 5 min), and resuspended in 10 mL complete DMEM. Cells were counted and seeded at 1 × 10^5^ cells/mL onto collagen-coated plates.

Pools of four siRNAs targeting GHR or non-targeting controls (Horizon-Dharmacon) (final concentration 20 nM) were added to a mixture of OptiMEM (Gibco) and transfection reagent (DharmaFECT, 15 μl per 1 ml OptiMEM); the mixture was added to collagen-coated wells (200 μL/well) and incubated for 20 min at room temperature (RT). Freshly isolated primary hepatocytes (*n* = 6 in si-GHR; *n* = 6 in non-targeting controls) were added to each well (800 μL/well), and medium containing siRNA and transfection reagents was replaced after 24 h.

### Hepatocyte glucose production

At 48 h post-seeding, cells were incubated in serum-free medium for 4 h, washed twice with PBS, and incubated for 2 h with DMEM containing glutamine, sodium lactate, and sodium pyruvate, in the presence or absence of pCPT-cAMP (100 μM, Sigma) and/or 10 nM insulin. After 2 h, the supernatant was collected, centrifuged twice (200 × *g*, 3 min, 340 × *g*, 3 min); 50 μL of the supernatant were used for glucose quantification (Thermo). The attached hepatocytes were washed once with PBS and frozen in −80 °C.

### RNA extraction and gene expression

RNA from primary hepatocytes and liver tissue was extracted using TRIzol reagent using the manufacturer’s protocol. cDNA was synthesized using random hexamers (High-Capacity cDNA Reverse Transcription Kit, ThermoFisher-Applied Biosystems). Real-time quantitative PCR was performed using SYBR green qPCR Supermix (Biorad) and QuantStudio 6 (Applied Biosystems). Primers are provided in Supplementary Table 1. Gene expression was quantified using the ddCt method.

### Reporting summary

Further information on research design is available in the Nature Research Reporting Summary linked to this article.

## Supplementary information

Supplementary Information

Description of Additional Supplementary Files

Supplementary Data 1

Supplementary Dataset 2

Supplementary Dataset 3

Reporting Summary

## Data Availability

The SOMAscan proteomics and clinical data generated in this study have been deposited in the Gene Expression Omnibus database under accession code GSE122279. The metabolomics data are provided in Supplementary Data 3. SOMAscan proteomics, metabolomics, clinical data, and the R/Bioconductor^85^ code to reproduce this article’s main results are publicly available on GitHub at https://github.com/jdreyf/slimm-t2d-omics and at Zenodo at 10.5281/zenodo.5485746^90^. A Source Data file is included. All remaining data generated or analyzed during this study are included in this published article (and its supplementary information files).

## Source data

Source Data

## Code availability

The Hitman R package is available at https://github.com/jdreyf/Hitman, which depends on the ezlimma package (https://github.com/jdreyf/ezlimma). We generated plots from its companion package, ezlimmaplot (https://github.com/jdreyf/ezlimmaplot). SOMAscan proteomics, metabolomics, clinical metadata, and the R/Bioconductor^85^ code to reproduce this article’s main results are available on GitHub at https://github.com/jdreyf/slimm-t2d-omics and at Zenodo at 10.5281/zenodo.5485746^90^. To reproduce the headline results with one click even when software versions of dependencies change, data and code have also been deposited in a Code Ocean capsule at 10.24433/CO.9548519.v2, which is open, exportable, reproducible, and interoperable.
