# Supplementary material for: High-throughput mediation analysis of human proteome and metabolome identifies mediators of post-bariatric surgical diabetes control
Source: Nat Commun. 2021 Nov 29;12:6951. doi: 10.1038/s41467-021-27289-2 (PMC8630169; doi:10.1038/s41467-021-27289-2)
Supplement: Supplementary file 1 — Supplementary Information [file 41467_2021_27289_MOESM1_ESM.pdf]

Supplementary Information Items

Supplementary Note 1. Hitman algorithm and validation. Technical description, mathematical justification, and simulations of Hitman and its simplified version without Limma.

Supplementary Figures 1-4.

Supplementary Table 1. PCR Primers.

References

Note S1. High-throughput mediation analysis (Hitman)

Model

As discussed in the manuscript, we need to know a priori whether or not our treatment or exposure (E) changes the outcome (Y) and in what direction. We then test whether or not the observed causal effect of the exposure on the outcome in our study is significant and is in the direction known a priori. We represent this potential causal effect as $E \longrightarrow Y$. Lack of an arrow indicates no causal effect,[1] so the model assumes that Y does not affect E (as occurs when E is randomized).

The test of $E \longrightarrow Y$ possibly includes covariates, but does not include the mediators. Our unmediated model (without covariates or error terms) is $E \xrightarrow{\lambda_1} Y$, where the structural parameter λ_1 represents the *total effect* (http://davidakenny.net/cm/mediate.htm). The corresponding structural equation model (SEM), with optional covariate X. If there are multiple covariates per sample, X is a vector per sample.

$$Y = \lambda_0 + \lambda_1 E + \lambda_2 X + U_{Y'}$$

The direction of the observed total effect is the sign of $\hat{\lambda}_1$, which is the estimate of λ_1. $\text{sgn}(\lambda_1)$ is predefined, and we should have $\text{sgn}(\hat{\lambda}_1) = \text{sgn}(\lambda_1)$. We test the null hypothesis $\lambda_1 = 0$, and if it is significant and $\text{sgn}(\hat{\lambda}_1) = \text{sgn}(\lambda_1)$, we infer that E causally affected Y. Once we have a causal effect, we can move on to Hitman to identify which analytes may be mediating it.

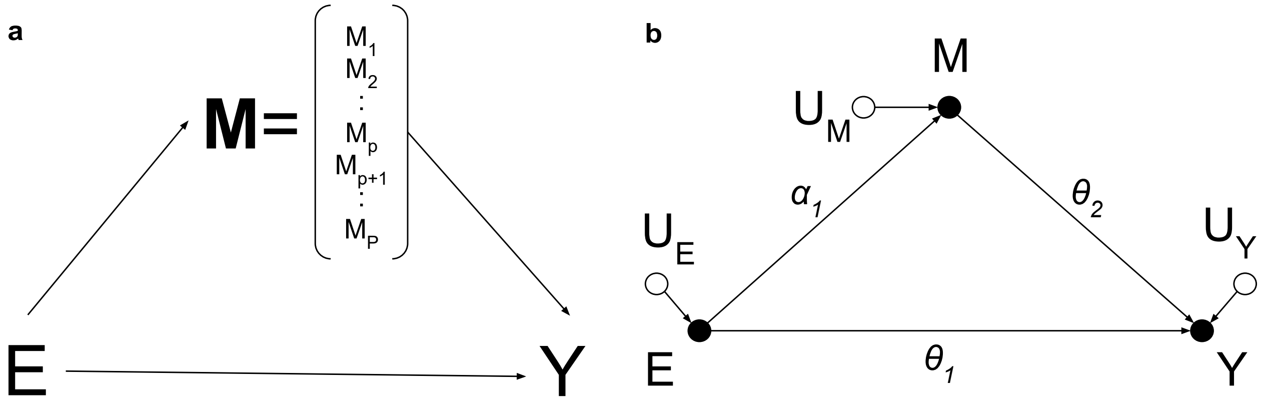

Our causal models including the effects of mediators (M) are shown in the figure above, which does not include covariates, and where error terms are suppressed in panel a. In panel a, we have $E \longrightarrow \mathbf{M} \longrightarrow Y$, with many mediators. Our causal mediation model per mediator is shown in panel b. We are then interested in the *mediation effect* (or *indirect effect*), represented by $\alpha_1\theta_2$. θ_1 is the *direct effect* per mediator. As lack of an arrow indicates no causal effect, this model assumes that neither M nor Y affect E (as occurs when E is randomized), and that Y does not affect M. Hitman does not test these assumptions.

Our model in panel a is based on a biological system, such as a cell, where an intervention (or exposure) like a gene knockout can have effects across all activities in the cell. The number of potential mediators of the exposure is P, where P is astronomically large. In an omics experiment, we measure p (with p much smaller than P, $p << P$) analytes across n samples, with $n << p$. The P mediators in Figure 1a have

unknown relationships among themselves and on the outcome. Ideally, we would identify these relationships so that we can account for them, but with few samples and an astronomical number of mostly unmeasured mediators, we do not attempt that here, and instead pursue an exploratory, one-at-a-time analysis of our measured mediators, so that we can identify which mediators to pursue with further studies.

Our linear SEM per mediator with covariate X is:

$$E = \nu_0 + \nu_1 X + U_E \tag{1}$$
$$M = \alpha_0 + \alpha_1 E + \alpha_2 X + U_M \tag{2}$$
$$Y = \theta_0 + \theta_1 E + \theta_2 M + \theta_3 X + U_Y \tag{3}$$

where U terms represent omitted factors that explain finite sources of variation. The SEM's coefficients represent finite structural parameters estimable from the data. These terms must be mutually independent for the validity of mediation analyses. When we randomize E, we know that U_E is independent of the other U's. However, U_M is often dependent with U_Y, due to another mediator (likely unmeasured) affecting both M and Y. So, "theoretical knowledge must be invoked to identify the sources of these correlations and control for common causes (so called 'confounders') of M and Y whenever they are observable".[2] Such confounders can be included in X. When X is a vector per sample, ν_1, α_2, and θ_3 are vectors.

Hitman is based on the causal steps approach. One variant of the causal steps approach is the joint significance test. Other variants of the causal steps approach test that the exposure affects the outcome, but the joint significance test does not[3][4]. The joint significance test has been shown to have more power than the product method and to control its false positive rate, because it is an intersection-union test.[5] The joint significance test calls as significant "consistent" mediators, which carry the causal effect, but it also calls as significant "inconsistent" mediators, which suppress the causal effect. However, the causal effect of the exposure on the outcome provides prior evidence and motivates the search only for consistent mediators. For example, if E increases Y (i.e. an increase in E increases Y), then we would want consistent mediators M such that E increases M and M increases Y, or such that E decreases M and M decreases Y. Whereas the joint significance test might find as significant an inconsistent mediator where E increases M and M decreases Y.[4] We address this in Hitman.

For each mediator (M), the null hypothesis is that E has no effect on M ($\alpha_1 = 0$); or that M has no effect on Y ($\theta_2 = 0$); or that the direction of mediation, $\text{sgn}(\alpha_1\theta_2)$, is not consistent with the direction of the total effect, $\text{sgn}(\lambda_1)$, with sgn being the sign function,

$$\text{sgn}(x) = \begin{cases} -1, & \text{if } x < 0 \\ 0, & \text{if } x = 0 \\ 1, & \text{if } x > 0 \end{cases}$$

The alternative hypothesis is that E affects M and M affects Y, and that the direction of $E \longrightarrow M$ and $M \longrightarrow Y$ are consistent with that of the total effect of E on Y. This tests if M explains at least some of the dependence between E and Y; M need not explain all of the dependence, but it could, in which case we have *complete mediation* and the direct effect vanishes.

Hitman implements tests with high-throughput mediators using the R/Bioconductor linear modeling package Limma.[6] Limma models the variance of features (e.g. proteins or metabolites) with an empirical Bayesian method, which exploits information about shared technical variance between features for improved power, especially when the sample size is small.[6] To model variance of feature abundance in linear modeling, Limma models feature abundance as the dependent variable.

Workflow

Assumptions

A1. E is randomized (given X, if X is provided)
A2. M and Y follow the linear SEM in equations 2 and 3
A3. U_E, U_M, and U_Y are mutually independent
A4. $U_M \overset{i.i.d.}{\sim} N$ and $U_Y \overset{i.i.d.}{\sim} N$, each with finite means and finite, positive variances
A5. $\text{sgn}(\lambda_1) = \text{sgn}\left(\hat{\lambda}_1\right)$ with $\hat{\lambda}_1 \neq 0$

Before Hitman

We need to ensure that the assumptions hold. Herein we describe and validate Hitman/Lotman for the simple case where the SEM is linear and M and Y being normally distributed, but it is straightforward to extend our approach to E being randomized given propensity scores; the value of M being associated with its variance, as happens with RNA-seq data, which Limma accommodates with weights;[7] and Y being modeled by a generalized linear model, such as Y following a binomial distribution.

The assumption that most differentiates Hitman from other mediation methods is A5. To ensure A5 holds, we pre-define $\text{sgn}(\lambda_1)$ and then we test the total effect λ_1 with $H_0 : \lambda_1 = 0$ against $H_a : \lambda_1 \neq 0$. This test must be significant and the observed causal effect must be in the same direction as previously known. If the test is not significant, we cannot be confident in the direction of the total effect in our study. Examples where A5 would not hold is if surgery did not cause a significant change in HbA1c or if surgery worsened HbA1c, which would contradict prior knowledge. If this pre-test fails, then STOP, because Hitman would assign all mediators a p-value of one, which is not likely to be helpful.

Hitman

For each mediator M:

1. Measure effect of E and M using equation 2. Here and later, if X is absent, remove its term. If X is vector-valued per sample, α_2 is a vector. Test $\alpha_1 = 0$ in Limma and define the resulting p-value p_1.

2. Measure association of M and Y given E (and possibly X) using equation 3. We would like to test $\theta_2 = 0$ using Limma. To model the variance of M with empirical Bayesian methods, we need to make M the dependent variable. So we use an approach similar to partial correlation.

    i. Estimate the residuals of $Y = \lambda_0 + \lambda_1 E + \lambda_2 X + e$ as e_Y.
    ii. Estimate the residuals of $M = \alpha_0 + \alpha_1 E + \alpha_2 X + e$ as e_M.
    iii. From the linear model $e_M = \theta_2 e_Y + \epsilon$, test $\theta_2 = 0$ in Limma and define the resulting p-value p_2.

Now define

$$S = \begin{cases} 0, & \text{if } \text{sgn}(\alpha_1\theta_2) \neq \text{sgn}(\lambda_1) \\ 1, & \text{if } \text{sgn}(\alpha_1\theta_2) = \text{sgn}(\lambda_1) \end{cases} \tag{4}$$

and

$$\hat{S} = \begin{cases} 0, & \text{if } \text{sgn}\left(\hat{\alpha}_1\hat{\theta}_2\right) \neq \text{sgn}\left(\hat{\lambda}_1\right) \\ 1, & \text{if } \text{sgn}\left(\hat{\alpha}_1\hat{\theta}_2\right) = \text{sgn}\left(\hat{\lambda}_1\right). \end{cases}$$

3. If $\hat{S} = 1$, then the direction of total effect agrees with that of $E \longrightarrow M \longrightarrow Y$, and the final p-value $= \frac{\max\{p_1, p_2\}}{2}$. Otherwise, the direction of the indirect effect is inconsistent with that of the total effect, and the final p-value $= 1$. The intuition behind the test is that knowing λ_1 allows for one-sided testing of the indirect effect.

To account for testing multiple mediators, false discovery rates (FDRs) or family-wise error rates (FWERs) can be calculated from the mediator p-values.

Simplified Hitman without Limma: Lotman

If we follow the Hitman workflow but apply linear regression models without Limma, in step 2 we test $\theta_2 = 0$ from equation 3 directly. This method could be used when Limma is not appropriate, such as for low-throughput data, so we term it Low-throughput mediation analysis (*Lotman*), and it is available in the R package `Hitman`.

Mathematical proof

Here, we consider Hitman theoretically to show that it controls its false positive rate. Hitman's use of the partial correlation approach in step 2 is a valid alternative to multiple regression, and incorporating Limma maintains control of the false positive rate, because Limma is theoretically sound[8] and empirically validated.[6] We do not consider these aspects theoretically here, although Hitman controls its false positive rate in simulations below, providing evidence that these aspects do not corrupt Hitman's validity. Hitman without these aspects is Lotman, so we consider the validity of both methods by essentially considering Lotman, but we point out where results with Limma would differ. Lotman applies tests in steps 1 and 2 with ordinary least squares (OLS), which provides unbiased estimates with valid p-values.

For a single mediator, the null and alternative hypotheses are:

$$H_0: \ \alpha_1 = 0 \cup \theta_2 = 0 \cup S = 0$$
$$H_a: \ \alpha_1 \neq 0 \cap \theta_2 \neq 0 \cap S = 1.$$

where $\cap$ represents "and" and $\cup$ represents "or". The terms in H_a are not independent, because the definition of S depends on α_1, θ_2, and λ_1 in equation 4. We have rejected the null hypothesis that $\lambda_1 = 0$ before applying Hitman, so $\hat{\lambda}_1 \neq 0$, and the results of this test are convincing, so we assume that there is a true causal effect in the direction observed, i.e. $\operatorname{sgn}(\lambda_1) = \operatorname{sgn}\left(\hat{\lambda}_1\right) \neq 0$. Without loss of generality, we consider $\operatorname{sgn}(\lambda_1) = \operatorname{sgn}\left(\hat{\lambda}_1\right) = 1$. Thus, from equation 4, $S = 1 \implies \operatorname{sgn}(\alpha_1\theta_2) \neq 0 \implies \alpha_1 \neq 0 \cap \theta_2 \neq 0$, with $\implies$ representing "implies". By definition, $H_a \implies S = 1$. Consequently,

$$S = 1 \iff H_a. \tag{5}$$

Thus, $S = 0 \implies H_0$. Both $\alpha_1 \implies S = 0$ and $\theta_2 \implies S = 0$, so $H_0 \implies S = 0$:

$$S = 0 \iff H_0. \tag{6}$$

From equation 4, $S = 0 \cap \operatorname{sgn}(\lambda_1) = 1 \implies \operatorname{sgn}(\alpha_1\theta_2) \neq 1$. We can see from the Hitman algorithm and hypotheses that α_1 and θ_2 are treated symmetrically, so without loss of generality, we consider the null hypothesis space,

$$H_0^\star: \alpha_1 \leq 0 \cap \theta_2 \geq 0 \cap \lambda_1 > 0$$

where by assumption A5 we also have $\hat{\lambda}_1 > 0$. To demonstrate that Hitman yields valid p-values, we need to show that Hitman p-values, p_{HM}, obey $P(p_{HM} \leq u | H_0^\star, \hat{\lambda}_1 > 0) \leq u$ for each $0 \leq u \leq 1$. Hitman is partially based on the joint significance test, and it has been shown that the joint significance p-values (p_{JS}) satisfy this property.[5] Let $p_{JS} = \max\{p_1, p_2\}$. Then, under $H_0^\star$ and $\hat{\lambda}_1 > 0$,

$$\begin{aligned} p_{HM} &= \frac{p_{JS}}{2}\hat{S} + (1 - \hat{S}) \\ &= \begin{cases} \frac{p_{JS}}{2}, & \text{if } \hat{S} = 1 \\ 1, & \text{if } \hat{S} = 0. \end{cases} \end{aligned} \tag{7}$$

where $0 \leq \frac{p_{JS}}{2} \leq \frac{1}{2}$. We can immediately see for $u = 1$ that $P(p_{HM} \leq 1) \leq 1$, and for $u = 0$, $P(p_{HM} = 0) = 0$, because p_{HM} is continuous over $\left[0, \frac{1}{2}\right]$.

We now consider how p_1 and p_2 are calculated. We know by assumption A4 that $0 < \sigma_{\alpha_1}, \sigma_{\theta_2} < \infty$. For arbitrary sample size N (sufficient to estimate parameters and variances) and full rank covariate matrix X of rank k, using OLS we have $T_1 := (\hat{\alpha}_1 - \alpha_1)/\hat{\sigma}_{\hat{\alpha}_1} = \tilde{\alpha}_1 - \frac{\alpha_1}{\hat{\sigma}_{\hat{\alpha}_1}} \sim t_{N-k-1}$, for $\tilde{\alpha}_1 := \frac{\hat{\alpha}_1}{\hat{\sigma}_{\hat{\alpha}_1}}$, where $:=$ refers to "defined by," and $T_2 := (\hat{\theta}_2 - \theta_2)/\hat{\sigma}_{\hat{\theta}_2} = \tilde{\theta}_2 - \frac{\theta_2}{\hat{\sigma}_{\hat{\theta}_2}} \sim t_{N-k-2}$, for $\tilde{\theta}_2 := \frac{\hat{\theta}_2}{\hat{\sigma}_{\hat{\theta}_2}}$. Whereas if we consider the asymptotic case or the actual instead of the estimated standard deviation, T_1 and T_2 follow the standard normal distribution with density ϕ and cumulative distribution function Φ. The degrees of freedom of the t-statistics will be increased with Limma's empirical Bayesian variance estimate, especially when $N - k$ is small.

Instead of relying on the t-statistics converging to the normal distribution asymptotically, our mathematical proof extends to small sample sizes, so we account for the two t-distributions' different degrees of freedom. We define the T_1 as having cumulative distribution function F_1 and probability density function f_1, whereas T_2 as having cumulative distribution function F_2 and probability density function f_2. To explain properties that apply to t-distributions (i.e. independent of their degrees of freedom, so including the standard normal distribution as a special case), we use F or f with subscripts suppressed. For example, we use below the properties of the t-distribution that $F(-x) = 1 - F(x)$ and $F^{-1}(1 - x) = -F^{-1}(x)$. We also suppress subscripts for the joint distribution of $\hat{\alpha}_1$ and $\hat{\theta}_2$.

In steps 1 and 2 of Hitman we test $\alpha_1 = 0$ and $\theta_2 = 0$ with estimates $\tilde{\alpha}_1 = a$ and $\tilde{\theta}_2 = t$ under $H_0^\star$ and $\hat{\lambda}_1 > 0$ by assumption A5. Then $p_1 = 2F_1(-|a|)$ and $p_2 = 2F_2(-|t|)$. So,

$$\frac{p_{JS}}{2} = \begin{cases} F_1(-|a|), & \text{if } F_1(-|a|) \geq F_2(-|t|) \\ F_2(-|t|), & \text{otherwise.} \end{cases} \tag{8}$$

Overview of mathematical proof

We have shown above that $P(p_{HM} \leq u | H_0^\star, \hat{\lambda}_1 > 0) \leq u$ for $u = 0$ and for $u = 1$, so we must show this for $0 < u < 1$. Equation 7 says that we need to consider $P(\hat{S} = 1 | H_0^\star, \hat{\lambda}_1 > 0)$, so our next step is to examine $\max P(\hat{S} = 1 | H_0^\star, \hat{\lambda}_1 > 0)$, where we show that $P(\hat{S} = 1 | H_0^\star, \hat{\lambda}_1 > 0) \leq \frac{1}{2}$. This bound allows us to immediately show that $P(p_{HM} \leq u | H_0^\star, \hat{\lambda}_1 > 0) \leq u$ for $u \geq \frac{1}{2}$.

However, the case of $u < \frac{1}{2}$ is more challenging, so we tackle it in several steps. First we show that $P(p_{HM} \leq u | H_0^\star, \hat{\lambda}_1 > 0) \leq u$ when either $\alpha_1 = 0$ or $\theta_2 = 0$, which is on the boundary of the space $\alpha_1 \times \theta_2 \mid H_0^\star, \hat{\lambda}_1 > 0$. We then evaluate the false positive rate over the full space $\alpha_1 \times \theta_2 \mid H_0^\star, \hat{\lambda}_1 > 0$. To find the optima of this space, we calculate its derivative and identify where the derivative is zero. We then calculate the values on the optima and on the boundary. We find that the maxima of $P(p_{HM} \leq u | H_0^\star, \hat{\lambda}_1 > 0)$ is on the boundary, where we know $P(p_{HM} \leq u | H_0^\star, \hat{\lambda}_1 > 0) \leq u$, which completes the proof.

Maximizing probability estimated direction is consistent

Under $H_0^\star$ we also have $\hat{\lambda}_1 > 0$. However we suppress the notation of conditioning on these variables for brevity in the following equation. We also have $\hat{\alpha}_1 \perp\!\!\!\perp \hat{\theta}_2$.[9]

$$
\begin{aligned}
&P(\hat{S}=1) \\
&= \int_{\hat{\alpha}_1^\dagger=-\infty}^{\infty}\int_{\hat{\theta}_2^\dagger=-\infty}^{\infty} P\Big(\hat{S}=1\big|\hat{\alpha}_1=\hat{\alpha}_1^\dagger,\hat{\theta}_2=\hat{\theta}_2^\dagger\Big)\phi\Big(\frac{\hat{\alpha}_1^\dagger-\alpha_1}{\sigma_{\hat{\alpha}_1}}\Big)\phi\Big(\frac{\hat{\theta}_2^\dagger-\theta_2}{\sigma_{\hat{\theta}_2}}\Big)d\hat{\theta}_2^\dagger d\hat{\alpha}_1^\dagger \\
&= \int_{\hat{\alpha}_1^\dagger=-\infty}^{0}\int_{\hat{\theta}_2^\dagger=-\infty}^{0} \phi\Big(\frac{\hat{\alpha}_1^\dagger-\alpha_1}{\sigma_{\hat{\alpha}_1}}\Big)\phi\Big(\frac{\hat{\theta}_2^\dagger-\theta_2}{\sigma_{\hat{\theta}_2}}\Big)d\hat{\theta}_2^\dagger d\hat{\alpha}_1^\dagger + \int_{\hat{\alpha}_1^\dagger=0}^{\infty}\int_{\hat{\theta}_2^\dagger=0}^{\infty} \phi\Big(\frac{\hat{\alpha}_1^\dagger-\alpha_1}{\sigma_{\hat{\alpha}_1}}\Big)\phi\Big(\frac{\hat{\theta}_2^\dagger-\theta_2}{\sigma_{\hat{\theta}_2}}\Big)d\hat{\theta}_2^\dagger d\hat{\alpha}_1^\dagger \\
&\quad \text{because } P\Big(\hat{S}=1\big|\hat{\alpha}_1=\hat{\alpha}_1^\dagger,\hat{\theta}_2=\hat{\theta}_2^\dagger,H_0^\star,\hat{\lambda}_1>0\Big) = \begin{cases}1, & \text{if } \hat{\alpha}_1^\dagger * \hat{\theta}_2^\dagger > 0,\\ 0, & \text{otherwise,}\end{cases} \\
&= \int_{\hat{\alpha}_1^\dagger=-\infty}^{0}\phi\Big(\frac{\hat{\alpha}_1^\dagger-\alpha_1}{\sigma_{\hat{\alpha}_1}}\Big)d\hat{\alpha}_1^\dagger\int_{\hat{\theta}_2^\dagger=-\infty}^{0}\phi\Big(\frac{\hat{\theta}_2^\dagger-\theta_2}{\sigma_{\hat{\theta}_2}}\Big)d\hat{\theta}_2^\dagger + \int_{\hat{\alpha}_1^\dagger=0}^{\infty}\phi\Big(\frac{\hat{\alpha}_1^\dagger-\alpha_1}{\sigma_{\hat{\alpha}_1}}\Big)d\hat{\alpha}_1^\dagger\int_{\hat{\theta}_2^\dagger=0}^{\infty}\phi\Big(\frac{\hat{\theta}_2^\dagger-\theta_2}{\sigma_{\hat{\theta}_2}}\Big)d\hat{\theta}_2^\dagger \qquad (9)\\
&= \lim_{\psi\to\infty}\Big[\Phi\Big(-\frac{\alpha_1}{\sigma_{\hat{\alpha}_1}}\Big)-\Phi\Big(\frac{-\psi-\alpha_1}{\sigma_{\hat{\alpha}_1}}\Big)\Big]\Big[\Phi\Big(-\frac{\theta_2}{\sigma_{\hat{\theta}_2}}\Big)-\Phi\Big(\frac{-\psi-\theta_2}{\sigma_{\hat{\theta}_2}}\Big)\Big] \\
&\quad + \Big[\Phi\Big(\psi-\frac{\alpha_1}{\sigma_{\hat{\alpha}_1}}\Big)-\Phi\Big(-\frac{\alpha_1}{\sigma_{\hat{\alpha}_1}}\Big)\Big]\Big[\Phi\Big(\frac{\psi-\theta_2}{\sigma_{\hat{\theta}_2}}\Big)-\Phi\Big(-\frac{\theta_2}{\sigma_{\hat{\theta}_2}}\Big)\Big] \\
&= 2\Phi\Big(-\frac{\alpha_1}{\sigma_{\hat{\alpha}_1}}\Big)\Phi\Big(-\frac{\theta_2}{\sigma_{\hat{\theta}_2}}\Big)+1-\Phi\Big(-\frac{\alpha_1}{\sigma_{\hat{\alpha}_1}}\Big)-\Phi\Big(-\frac{\theta_2}{\sigma_{\hat{\theta}_2}}\Big), \\
&\quad\quad \text{because } \lim_{\psi\to\infty}\Phi(\psi)=1 \text{ and } \lim_{\psi\to\infty}\Phi(-\psi)=0.
\end{aligned}
$$

Then, $\frac{1}{2} \leq \Phi\Big(-\frac{\alpha_1}{\hat{\sigma}_{\hat{\alpha}_1}}\Big) \leq 1$ and $0 \leq \Phi\Big(-\frac{\theta_2}{\hat{\sigma}_{\hat{\theta}_2}}\Big) \leq \frac{1}{2}$.

To find the upper bound of equation 9, we maximize equation 9 as $\omega(v_1, v_2)$ with $v_1 = \Phi\Big(-\frac{\alpha_1}{\hat{\sigma}_{\hat{\alpha}_1}}\Big)$ and $v_2 = \Phi\Big(-\frac{\theta_2}{\hat{\sigma}_{\hat{\theta}_2}}\Big)$. Our bilinear program is

$$
\begin{aligned}
\max_{v_1,v_2} \quad & 2v_1v_2 - v_1 - v_2 + 1 \\
\text{subject to} \quad & \\
& \frac{1}{2} \leq v_1 \leq 1 \\
& 0 \leq v_2 \leq \frac{1}{2}
\end{aligned}
$$

which cannot be solved by convex programming. However, we can examine where one of v_1 or v_2 is fixed, giving a linear program. Consider the linear program for any fixed $v_1 \in (\frac{1}{2}, 1]$:

$$
\begin{aligned}
\operatorname*{argmax}_{v_2} \quad & (2v_1 - 1)v_2 + 1 - v_1 \\
\text{subject to} \quad & \\
& 0 \leq v_2 \leq \frac{1}{2}
\end{aligned}
$$

yields $v_2^* = \frac{1}{2}$. Whereas, $v_2 \in [0, \frac{1}{2}) \implies v_1^* = \frac{1}{2}$. So we have $v_1^* = \frac{1}{2} \cup v_2^* = \frac{1}{2}$, and under the above constraints, $\max_{v_1,v_2} \omega(v_1, v_2) = \frac{1}{2}$. Transforming back to equation 9, $v_1 = \frac{1}{2} \implies \alpha_1 = 0$ and $v_2 = \frac{1}{2} \implies \theta_2 = 0$, so $\alpha_1^* = 0 \cup \theta_2^* = 0$. Consequently,

$$\max_{\alpha_1,\theta_2} \quad P(\hat{S}=1|\alpha_1 \le 0, \theta_2 \ge 0, \lambda_1 > 0, \hat{\lambda}_1 > 0) = \frac{1}{2}.$$

Case of $u \ge \frac{1}{2}$

A straightforward consequence of the bound on $P(\hat{S}=1)$ is for $\frac{1}{2} \le u < 1$. Under $H_0^\star$ and $\hat{\lambda}_1 > 0$, we see that

$$\begin{aligned} P(p_{HM} \le u) &= P(1 \le u|\hat{S}=0)P(\hat{S}=0) + P\left(\frac{p_{JS}}{2} \le u|\hat{S}=1\right)P(\hat{S}=1) \\ &= P(\hat{S}=1) \\ &\le \frac{1}{2}, \quad \text{as shown above,} \\ &\le u. \end{aligned} \tag{10}$$

False positive rate on the boundary

We next consider the special case of $\alpha_1 \times \theta_2$ on its boundary under the null. The boundary of $H_0^\star$ for $\alpha_1 \times \theta_2$ is $\alpha_1 = 0 \cup \theta_2 = 0$. A property of the boundary is that it corresponds to the null hypothesis of the joint significance test, where $P(p_{JS} \le u) \le u$. We can use this fact to show p_{HM} control its false positive rate under $H_0^\star$ on the boundary for $0 < u < \frac{1}{2}$:

$$\begin{aligned} P(p_{HM} \le u|\alpha_1 = 0 \cup \theta_2 = 0) =& P\Big(\frac{p_{JS}}{2} \le u|\alpha_1 = 0 \cup \theta_2 = 0, \hat{S}=1\Big)P(\hat{S}=1) \\ =& \frac{1}{2}P(p_{JS} \le 2u|\alpha_1 = 0 \cup \theta_2 = 0, \hat{S}=1) \\ \le& \frac{1}{2}2u \\ \le& u. \end{aligned} \tag{11}$$

In the special case $\alpha_1 = 0$ and $\theta_2 \to \infty$, $p_{JS} = p_1$ so:

$$\begin{aligned} \lim_{\theta_2 \to \infty} P(p_{HM} \le u|\alpha_1 = 0, \theta_2) &= \lim_{\theta_2 \to \infty} P\Big(\frac{p_1}{2} \le u|\alpha_1 = 0, \theta_2, \hat{S}=1\Big)P(\hat{S}=1) \\ &= \lim_{\theta_2 \to \infty} \frac{1}{2}P(p_1 \le 2u|\alpha_1 = 0, \theta_2, \hat{S}=1) \\ &= \frac{1}{2}2u \\ &= u. \end{aligned} \tag{12}$$

because $p_1 \sim U(0,1)$. Analogously, in the special case $\alpha_1 \to -\infty$ and $\theta_2 = 0$, $p_{JS} = p_2$, and we find that $P(p_{HM} \le u|\alpha_1 \to -\infty, \theta_2 = 0) = u$. So we see in these special cases that $P(p_{HM} \le u)$ reaches its maximum value of u.

When both parameters are zero,

$$
\begin{aligned}
P(p_{HM} \leq u|\alpha_1 = 0, \theta_2 = 0) =& P\Big(\frac{p_1}{2} \leq u, \frac{p_2}{2} \leq u|\alpha_1 = 0, \theta_2 = 0, \hat{S} = 1\Big)P(\hat{S} = 1) \\
=& \frac{1}{2}P\Big(\frac{p_1}{2} \leq u|\alpha_1 = 0, \hat{S} = 1\Big)P\Big(\frac{p_2}{2} \leq u|\theta_2 = 0, \hat{S} = 1\Big) \\
=& \frac{1}{2}P\Big(p_1 \leq 2u|\alpha_1 = 0, \hat{S} = 1\Big)P\Big(p_2 \leq 2u|\theta_2 = 0, \hat{S} = 1\Big) \\
=& \frac{1}{2}(2u)(2u) \\
=& 2u^2 \\
<& u, \quad \text{for } 0 < u < \frac{1}{2}.
\end{aligned} \tag{13}
$$

False positive rate in general

As above, under $H_0^\star$ we have $\hat{\lambda}_1 > 0$. We now define a density and cumulative distribution function of $\tilde{\alpha}_1$ that suppresses notation:

$$
\begin{aligned}
g_1(\tilde{\alpha}_1 = a) &= f_1\Big(a - \frac{\alpha_1}{\hat{\sigma}_{\hat{\alpha}_1}}\big|H_0^\star, \hat{\lambda}_1 > 0, \hat{\sigma}_{\hat{\alpha}_1}\Big) \\
G_1(\tilde{\alpha}_1 = a) &= F_1\Big(a - \frac{\alpha_1}{\hat{\sigma}_{\hat{\alpha}_1}}\big|H_0^\star, \hat{\lambda}_1 > 0, \hat{\sigma}_{\hat{\alpha}_1}\Big)
\end{aligned}
$$

where $\hat{\sigma}_{\hat{\alpha}_1} \perp\!\!\!\perp \alpha_1, \hat{\alpha}_1$. We have similar notation for $\tilde{\theta}_2$ with density and distribution having subscript 2. We determine the false positive rate for fixed $0 < u < \frac{1}{2}$ through the law of total probability,

$$
\begin{aligned}
&P(p_{HM} \leq u|H_0^\star, \hat{\lambda}_1 > 0, \hat{\sigma}_{\hat{\alpha}_1}, \hat{\sigma}_{\hat{\theta}_2}) \\
&= \int_{a=-\infty}^{\infty}\int_{t=-\infty}^{\infty} P(p_{HM} \leq u|\tilde{\alpha}_1 = a, \tilde{\theta}_2 = t, H_0^\star, \hat{\lambda}_1 > 0, \hat{\sigma}_{\hat{\alpha}_1}, \hat{\sigma}_{\hat{\theta}_2})g(\tilde{\alpha}_1 = a, \tilde{\theta}_2 = t)dtda \\
&= \int_{a=0}^{\infty}\int_{t=0}^{\infty} P(F_1(-a) \leq u, F_2(-t) \leq u)g_1(\tilde{\alpha}_1 = a)g_2(\tilde{\theta}_2 = t)dtda \\
&\quad + \int_{a=-\infty}^{0}\int_{t=-\infty}^{0} P(F_1(a) \leq u, F_2(t) \leq u)g_1(\tilde{\alpha}_1 = a)g_2(\tilde{\theta}_2 = t)dtda \\
&= \int_{a=0}^{\infty}\int_{t=0}^{\infty} P(-a \leq F_1^{-1}(u), -t \leq F_2^{-1}(u))g_1(\tilde{\alpha}_1 = a)g_2(\tilde{\theta}_2 = t)dtda \\
&\quad + \int_{a=-\infty}^{0}\int_{t=-\infty}^{0} P(a \leq F_1^{-1}(u), t \leq F_2^{-1}(u))g_1(\tilde{\alpha}_1 = a)g_2(\tilde{\theta}_2 = t)dtda \\
&= \int_{a=0}^{\infty}\int_{t=0}^{\infty} P(a \geq -F_1^{-1}(u))P(t \geq -F_1^{-1}(u))g_1(\tilde{\alpha}_1 = a)g_2(\tilde{\theta}_2 = t)dtda \\
&\quad + \int_{a=-\infty}^{0}\int_{t=-\infty}^{0} P(a \leq F_1^{-1}(u))P(t \leq F_2^{-1}(u))g_1(\tilde{\alpha}_1 = a)g_2(\tilde{\theta}_2 = t)dtda
\end{aligned} \tag{14}
$$

where for fixed values a, t and u, the probabilities $P(a \leq F_1^{-1}(u))$ and $P(t \geq -F_2^{-1}(u))$ resolve to zero or one and are independent because a and t are realizations of random variables, rather than being random variables themselves. That is,

$$
P(a \leq F_1^{-1}(u), t \leq F_2^{-1}(u)) = P(a \leq F_1^{-1}(u))P(t \leq F_2^{-1}(u)) = \begin{cases} 1, & \text{if } a \leq F_1^{-1}(u) \cap t \leq F_2^{-1}(u), \\ 0, & \text{otherwise.} \end{cases}
$$

We continue equation 14 to evaluate $P(p_{HM} \leq u | H_0^\star, \hat{\lambda}_1 > 0, \hat{\sigma}_{\hat{\alpha}_1}, \hat{\sigma}_{\hat{\theta}_2})$ but suppress conditioning on $H_0^\star, \hat{\lambda}_1 > 0, \hat{\sigma}_{\hat{\alpha}_1}, \hat{\sigma}_{\hat{\theta}_2}$:

$$
\begin{aligned}
&\int_{a=-F_1^{-1}(u)}^{\infty} g_1(\tilde{\alpha}_1 = a) da \int_{t=-F_2^{-1}(u)}^{\infty} g_2(\tilde{\theta}_2 = t) dt \\
&+ \int_{a=-\infty}^{F_1^{-1}(u)} g_1(\tilde{\alpha}_1 = a) da \int_{t=-\infty}^{F_2^{-1}(u)} g_2(\tilde{\theta}_2 = t) dt \\
=& \lim_{\psi \to \infty} \left[F_1\left(\psi - \frac{\alpha_1}{\hat{\sigma}_{\hat{\alpha}_1}}\right) - F_1\left(-F_1^{-1}(u) - \frac{\alpha_1}{\hat{\sigma}_{\hat{\alpha}_1}}\right) \right] \left[F_2\left(\psi - \frac{\theta_2}{\hat{\sigma}_{\hat{\theta}_2}}\right) - F_2\left(-F_2^{-1}(u) - \frac{\theta_2}{\hat{\sigma}_{\hat{\theta}_2}}\right) \right] \\
&+ \left[F_1\left(F_1^{-1}(u) - \frac{\alpha_1}{\hat{\sigma}_{\hat{\alpha}_1}}\right) - F_1\left(-\psi - \frac{\alpha_1}{\hat{\sigma}_{\hat{\alpha}_1}}\right) \right] \left[F_2\left(F_2^{-1}(u) - \frac{\theta_2}{\hat{\sigma}_{\hat{\theta}_2}}\right) - F_1\left(-\psi - \frac{\alpha_1}{\hat{\sigma}_{\hat{\alpha}_1}}\right) \right] \\
=& \left[1 - F_1\left(-F_1^{-1}(u) - \frac{\alpha_1}{\hat{\sigma}_{\hat{\alpha}_1}}\right) \right] \left[1 - F_2\left(-F_2^{-1}(u) - \frac{\theta_2}{\hat{\sigma}_{\hat{\theta}_2}}\right) \right] \\
&+ \left[F_1\left(F_1^{-1}(u) - \frac{\alpha_1}{\hat{\sigma}_{\hat{\alpha}_1}}\right) - 0 \right] \left[F_2\left(F_2^{-1}(u) - \frac{\theta_2}{\hat{\sigma}_{\hat{\theta}_2}}\right) - 0 \right] \\
=& F_1\left(F_1^{-1}(u) + \frac{\alpha_1}{\hat{\sigma}_{\hat{\alpha}_1}}\right) F_2\left(F_2^{-1}(u) + \frac{\theta_2}{\hat{\sigma}_{\hat{\theta}_2}}\right) \\
&+ F_1\left(F_1^{-1}(u) - \frac{\alpha_1}{\hat{\sigma}_{\hat{\alpha}_1}}\right) F_2\left(F_2^{-1}(u) - \frac{\theta_2}{\hat{\sigma}_{\hat{\theta}_2}}\right).
\end{aligned}
$$

When one of the parameters is on the boundary at zero, say $\alpha_1 = 0$, then equation 14 simplifies to

$$
u \left[F_2\left(F_2^{-1}(u) + \frac{\theta_2}{\hat{\sigma}_{\hat{\theta}_2}}\right) + F_2\left(F_2^{-1}(u) - \frac{\theta_2}{\hat{\sigma}_{\hat{\theta}_2}}\right) \right]
$$

and if also $\theta_2 \to \infty$, then in this special case,

$$
\lim_{\theta_2 \to \infty} P(p_{HM} \leq u | \alpha_1 = 0, \theta_2) = u\left[1 + 0\right] = u,
$$

which is the same result as equation 12, confirming our calculations.

Derivative of the false positive rate

We examine the derivative of equation 14 with respect to α_1 and θ_2 for $0 < u < \frac{1}{2}$ under $H_0^\star, \hat{\lambda}_1 > 0, \hat{\sigma}_{\hat{\alpha}_1}, \hat{\sigma}_{\hat{\theta}_2}$ to understand where the maxima may be:

$$
\frac{\partial P(p_{HM} \leq u)}{\partial \alpha_1} = \frac{1}{\hat{\sigma}_{\hat{\alpha}_1}} \left\{ f_1\left(F_1^{-1}(u) + \frac{\alpha_1}{\hat{\sigma}_{\hat{\alpha}_1}}\right) F_2\left(F_2^{-1}(u) + \frac{\theta_2}{\hat{\sigma}_{\hat{\theta}_2}}\right) - f_1\left(F_1^{-1}(u) - \frac{\alpha_1}{\hat{\sigma}_{\hat{\alpha}_1}}\right) F_2\left(F_2^{-1}(u) - \frac{\theta_2}{\hat{\sigma}_{\hat{\theta}_2}}\right) \right\}. \tag{15}
$$

We find that equation $\frac{\partial P(p_{HM} \leq u)}{\partial \alpha_1} = 0$ if $\alpha_1 = 0 \cap \theta_2 = 0$ or for any $\theta_2 \geq 0$ if $\alpha_1 \to -\infty$. Next,

$$
\frac{\partial P(p_{HM} \leq u)}{\partial \theta_2} = \frac{1}{\hat{\sigma}_{\hat{\theta}_2}} \left\{ F_1\left(F_1^{-1}(u) + \frac{\alpha_1}{\hat{\sigma}_{\hat{\alpha}_1}}\right) f_2\left(F_2^{-1}(u) + \frac{\theta_2}{\hat{\sigma}_{\hat{\theta}_2}}\right) - F_1\left(F_1^{-1}(u) - \frac{\alpha_1}{\hat{\sigma}_{\hat{\alpha}_1}}\right) f_2\left(F_2^{-1}(u) - \frac{\theta_2}{\hat{\sigma}_{\hat{\theta}_2}}\right) \right\}. \tag{16}
$$

And $\frac{\partial P(p_{HM} \leq u)}{\partial \theta_2} = 0$ if $\alpha_1 = 0 \cap \theta_2 = 0$ or for any $\alpha_1 \leq 0$ if $\theta_2 \to \infty$.

So off the boundary there is the optimum,

$$
\begin{aligned}
&= \lim_{\alpha_1 \to -\infty, \theta_2 \to \infty} F_1\left(F_1^{-1}(u) + \frac{\alpha_1}{\hat{\sigma}_{\hat{\alpha}_1}}\right) F_2\left(F_2^{-1}(u) + \frac{\theta_2}{\hat{\sigma}_{\hat{\theta}_2}}\right) \\
&\quad + F_1\left(F_1^{-1}(u) - \frac{\alpha_1}{\hat{\sigma}_{\hat{\alpha}_1}}\right) F_2\left(F_2^{-1}(u) - \frac{\theta_2}{\hat{\sigma}_{\hat{\theta}_2}}\right) \\
&= 0 * 1 + 1 * 0 \\
&= 0
\end{aligned}
$$

which is a minimum. Thus, the false positive rate is controlled off the boundary and on the boundary, so the false positive rate is controlled in general.

Example of inconsistent mediator

We provide a clear example of an inconsistent mediator in the figure below, which includes regression lines and 95% confidence interval bands. Source data is available at src_data_hm_toy.csv.

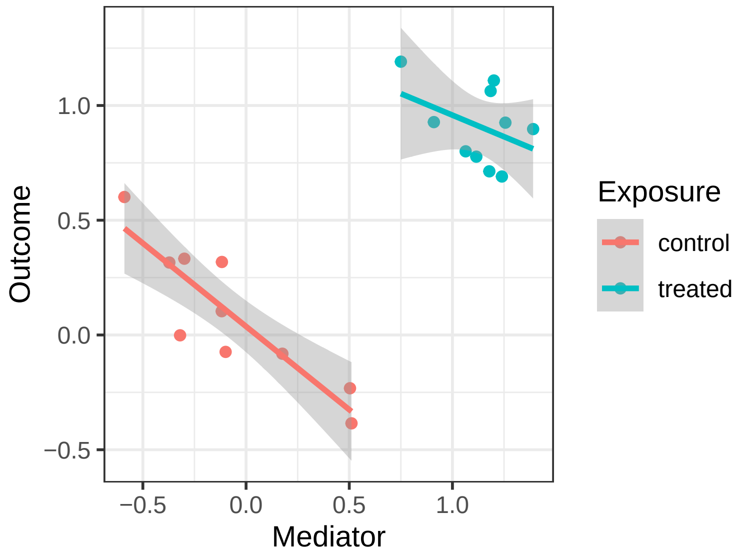

This figure is reminiscent of Simpson's Paradox.[10] The exposure increases the mediator and the exposure increases the outcome, so the direct effect is positive. But the mediator *decreases* the outcome, so the indirect effect is negative. Thus, we have inconsistent mediation, so Hitman conservatively estimates this mediator's p-value as 1, whereas the joint significance method would yield a p-value < 0.001 for this example.

Simulations following Barfield et al. (2017) & MacKinnon et al. (2002)

We validated our size and power following a simulation study.[11] This study's parameters were a subset of a previous simulation study (MacKinnon et al., 2002). Unlike the methods tested with these simulation scenarios, Hitman requires a predefined direction of the total effect, and includes a pre-test that the total

effect is significant and that its direction agrees with the observed total effect direction. It would seem unfair to provide Hitman the true direction of the total effect and this also wouldn't be sufficient to increase the chances that the total effect is significant. Instead, we could address this with a robust direct effect, so that the observed effect will tend to be significant and in the same direction as the true total effect. Although Barfield et al. (2017) set the direct effect to be "small", $\theta_1 = 0.14$, MacKinnon et al. (2002) included a scenario with a parameter that was "large" with value 0.59, so we chose to set $\theta_1 = 0.59$.

Like Barfield et al. (2017), we simulated data from $Y = \theta_0 + \theta_1 E + \theta_2 M + \theta_3 X + e_Y$, where M was simulated as $M = \alpha_0 + \alpha_1 E + \alpha_2 X + e_M$. (Note that our α corresponds to Barfield et al.'s β.) Again following Barfield et al. (2017), X and E and the error terms e_Y and e_M were simulated as independent standard normal variables; we set $\nu_0 = \nu_1 = 0$ and $\theta_0 = \theta_3 = \alpha_0 = \alpha_2 = 0.14$; and we simulated all combinations of θ_2 , $\alpha_1 \in$ (0, 0.14, 0.39), which correspond to effects of "zero", "small," and "medium" size, respectively (MacKinnon et al., 2002).

To account for the high-throughput data Hitman is designed to be applied to, we simulated other, null mediators as $M_i = \alpha_0 + \alpha_2 X + e_{M_i}$ for $i = 2, 3, ..., 10$, which are independent of the exposure and the outcome. These null mediators are utilized by Limma to estimate the shared technical variance, as would happen in an omics study. We do not test these other mediators.

We tested in what proportion of 10,000 simulations across 50 samples M achieved a p-value ≤ 0.05. Our results for these parameter values for Hitman, the joint significance test, and the mediate function from the R package mediation are shown in the table below.

α_1	θ_2	Hitman	Lotman	joint	mediate
0.00	0.00	0.006	0.005	0.002	0.002
0.00	0.14	0.013	0.011	0.006	0.005
0.14	0.00	0.016	0.013	0.008	0.007
0.00	0.39	0.040	0.041	0.036	0.030
0.39	0.00	0.049	0.040	0.038	0.028
0.14	0.14	0.064	0.055	0.023	0.015
0.14	0.39	0.208	0.199	0.108	0.103
0.39	0.14	0.211	0.195	0.111	0.127
0.39	0.39	0.685	0.681	0.539	0.574

A statistical test's size is the probability of falsely rejecting the null hypothesis, which is the probability of a false positive or a Type 1 error. All the methods here control their size, as they maintain a false positive rate less than 5%.

A statistical test's power is the probability that the test correctly rejects the null hypothesis when the alternative hypothesis is true, and it's inversely related to the probability of making a Type 2 error. To test Hitman's power, we look at the cases where both of θ_2 and α_1 are positive. Here, Hitman's power is greater than the other methods.

Hitman's power is similar to Lotman because we have a relatively large sample size. However, for a smaller sample size, we can see a larger difference between Hitman and Lotman. Here we show the same table, but for 15 samples.

α_1	θ_2	Hitman	Lotman	joint	mediate
0.00	0.00	0.007	0.004	0.002	0.002
0.00	0.14	0.011	0.007	0.004	0.008
0.14	0.00	0.010	0.007	0.003	0.003
0.00	0.39	0.023	0.017	0.008	0.013
0.39	0.00	0.029	0.018	0.013	0.015
0.14	0.14	0.019	0.012	0.005	0.006

α_1	θ_2	Hitman	Lotman	joint	mediate
0.14	0.39	0.050	0.032	0.012	0.024
0.39	0.14	0.064	0.042	0.016	0.026
0.39	0.39	0.161	0.118	0.052	0.066

Omics simulations

We also validated our methods in an omics setting with 500 analytes (which can interchangably be called "features" or "genes"; $g = 1, 2, ..., G$ with $G = 500$), where we address the multiplicity issue by controlling the false discovery rate (FDR). We simulated the data under 3 scenarios.

Scenario 1: Genes are independent and mediators are consistent

Scenario 2: Genes are dependent and mediators are consistent

Scenario 3: Genes are dependent and there are both consistent and inconsistent mediators

In all scenarios, we simulated datasets with sample sizes of N=15 or N=50 and either 1, 5, or 25 consistent mediators. The exposure often affects many analytes, so simulated that the exposure additionally affects 200 analytes with the same effect size that it affects mediators. However, these 200 analytes are not simulated to affect the outcome, so they are not mediators.

In scenario 3, for each consistent mediator there was also an inconsistent mediator of the same effect size. As above, we set $\theta_0 = \theta_3 = \alpha_0 = \alpha_2 = 0.14$, and here we set $\beta_1 = \theta_2 = 2$. To satisfy Hitman's requirement that the total effect be known a priori and observed in the dataset where Hitman is applied, we wanted $\theta_1 >> 2$ * number of mediators, so we set $\theta_1 = 10$ * number of mediators (including both consistent and inconsistent mediators).

We tested each gene for mediation using Hitman, Lotman, and the joint significance method. We corrected the p-values using the Benjamini-Hochberg method, thresholded the FDR at 15%, and calculated the number of potential mediators whose null is rejected, the proportion of true mediators whose null is rejected (power), and the proportion of false rejections among all rejections, which is the false discovery proportion (FDP). We conducted 1,000 such simulations and report the averages (means) of these statistics. Because FDR=E(FDP), the mean FDP is an estimate of the empirical FDR.

We still simulated following equation (1) here, with $E \sim N(0, 1)$, $X \sim N(0, 1)$, and $\nu_0 = \nu_1 = 0$. The matrix M with 500 rows and N columns is simulated as

$$M = \alpha_0 + \alpha_1 E + \alpha_2 X + U_M$$

where $\alpha_0, \alpha_1, \alpha_2 \in R^G$; $\alpha_{1g} = 1$ if gene g is a consistent or inconsistent mediator or is otherwise simulated to be affected by the exposure and $\alpha_{1g} = 0$ otherwise; and $U_M \sim N_G(0, \Sigma)$ with Σ a G-by-G positive definite matrix.

Our covariance matrix is derived from GTEx v8 RNA-seq gene read counts downloaded from https://gtexportal.org/home/datasets. We choose to focus on skeletal muscle tissue, since it had the most samples. We filtered out genes with low expression, applied TMM normalization,[12] accounted for measured covariates known to affect expression and inferred technical factors with SVA, like,[13] calculated the covariance matrix of 500 randomly sampled genes, and then scaled this matrix so that the median variance would be one. The script implementing this process is gtex_muscle_cov_subset_scaled.R and the processed matrix is at gtex_muscle_cov_subset_scaled.csv.

When genes are independent (scenario 1), Σ is the matrix whose diagonal elements are the same as the diagonal of the scaled covariance matrix, and all off-diagonal elements are zero. When genes are dependent (scenarios 2 & 3) Σ is the scaled covariance matrix.

Finally,

$$Y = \theta_0 + \theta_1 E + \sum_{g=1}^{G} \theta_{2g} M_g + \theta_3 X + U_Y$$

where $U_Y \sim N(0,1)$ and

$$\theta_{2g} = \begin{cases} -1, & \text{if gene g is an inconsistent mediator} \\ 0, & \text{if gene g is not a mediator} \\ 1, & \text{if gene g is a consistent mediator.} \end{cases}$$

For scenario 3, the joint significance method has power to identify both consistent and inconsistent mediators, whereas Hitman and Lotman only have power for consistent mediators, so we had a choice as to assess the joint significance method's power and FDR with respect to only consistent mediators (like Hitman and Lotman) or with respect to both types of mediators. We chose to show both types of assessments: one as "Joint signif consistent" and the other as "Joint signif both".

The omics simulation results in the figure below shows the FDR and power of the considered methods in the three scenarios. Our code implementing the simulations is at simulations_omics.Rmd and the CSV tables with the simulation output are in the folder validation whose file names include "sc1", "sc2", or "sc3" for scenarios 1, 2, and 3, respectively.

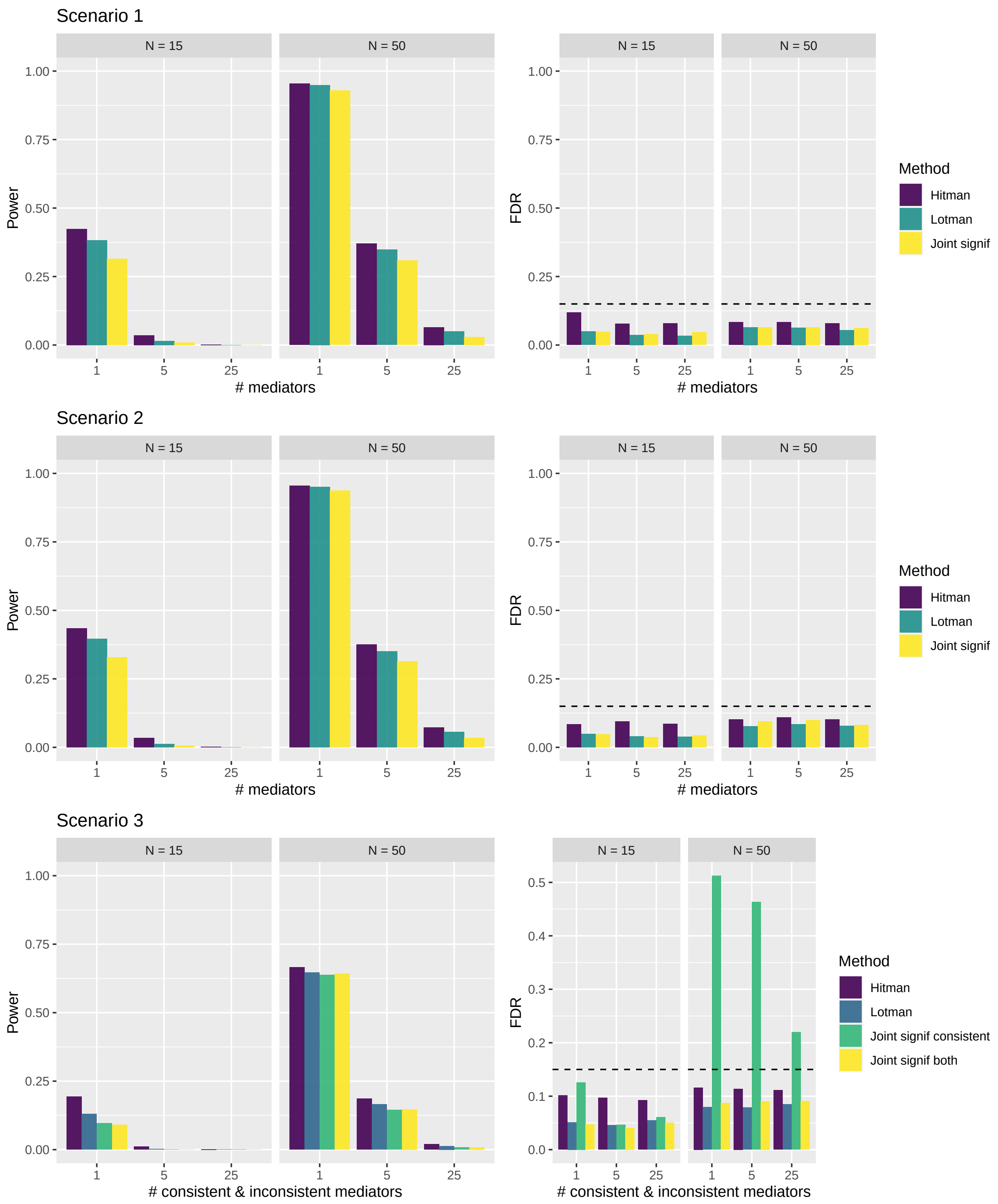
Scenario 1
N = 15
N = 50
Power
FDR
1.00
0.75
0.50
0.25
0.00
1
5
25
mediators
Method
Hitman
Lotman
Joint signif
Scenario 2
N = 15
N = 50
Power
FDR
mediators
Method
Hitman
Lotman
Joint signif
Scenario 3
N = 15
N = 50
Power
FDR
0.5
0.4
0.3
0.2
0.1
0.0
consistent & inconsistent mediators
Method
Hitman
Lotman
Joint signif consistent
Joint signif both

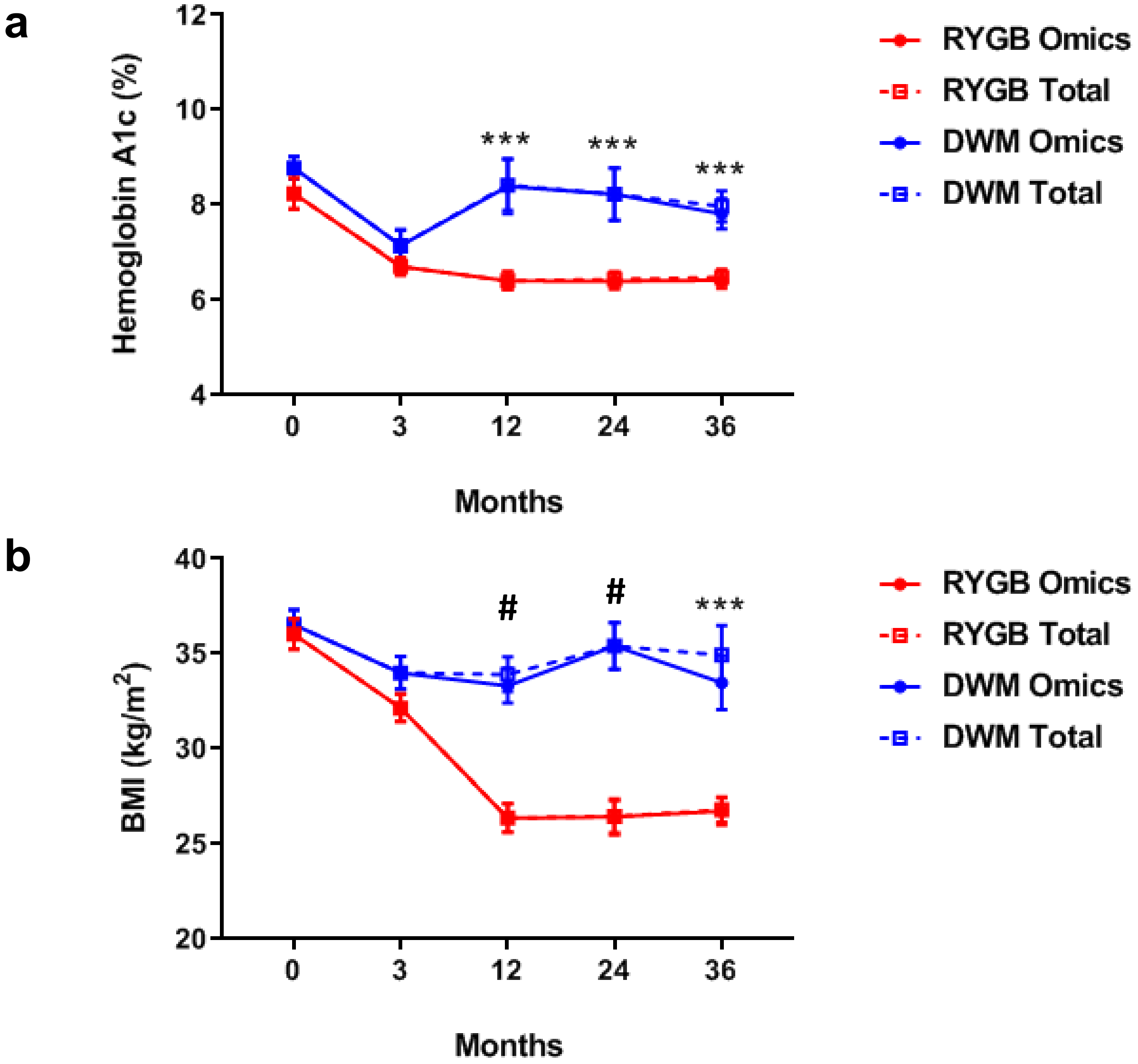

Figure S1. HbA1c and BMI for SLIMM-T2D cohort and those that had omics per time point. (a) HbA1c and **(b)** BMI for participants in the SLIMM-T2D cohort ("Total", squares) and those that had proteomics or metabolomics per time point ("Omics", circles). Data are reported as mean ± SEM. Data were analyzed by two-sided t-tests. For HbA1c and BMI, no significant difference was found between the Total and Omics groups within RYGB or within DWM at any time point. However, differences between RYGB and DWM per time point were found, with p values for omics subcohort as follows: (**a**) HbA1c: 12 months: ***=0.0015; 24 mo: ***=0.0012; 36 mo: ***=0.00046; (**b**) BMI: 12 mo: #<0.0001, 24 mo: #<0.0001; 36 mo: ***=0.00014. Samples with HbA1c were available from 19, 19, 19, 17, 16, and 15 participants from the RYGB arm, and 19, 19, 18, 11, 10, and 10 participants from the DWM arm at the baseline, 3, 12, 18, 24, and 36 month time points. Source data are provided as a Source Data file.

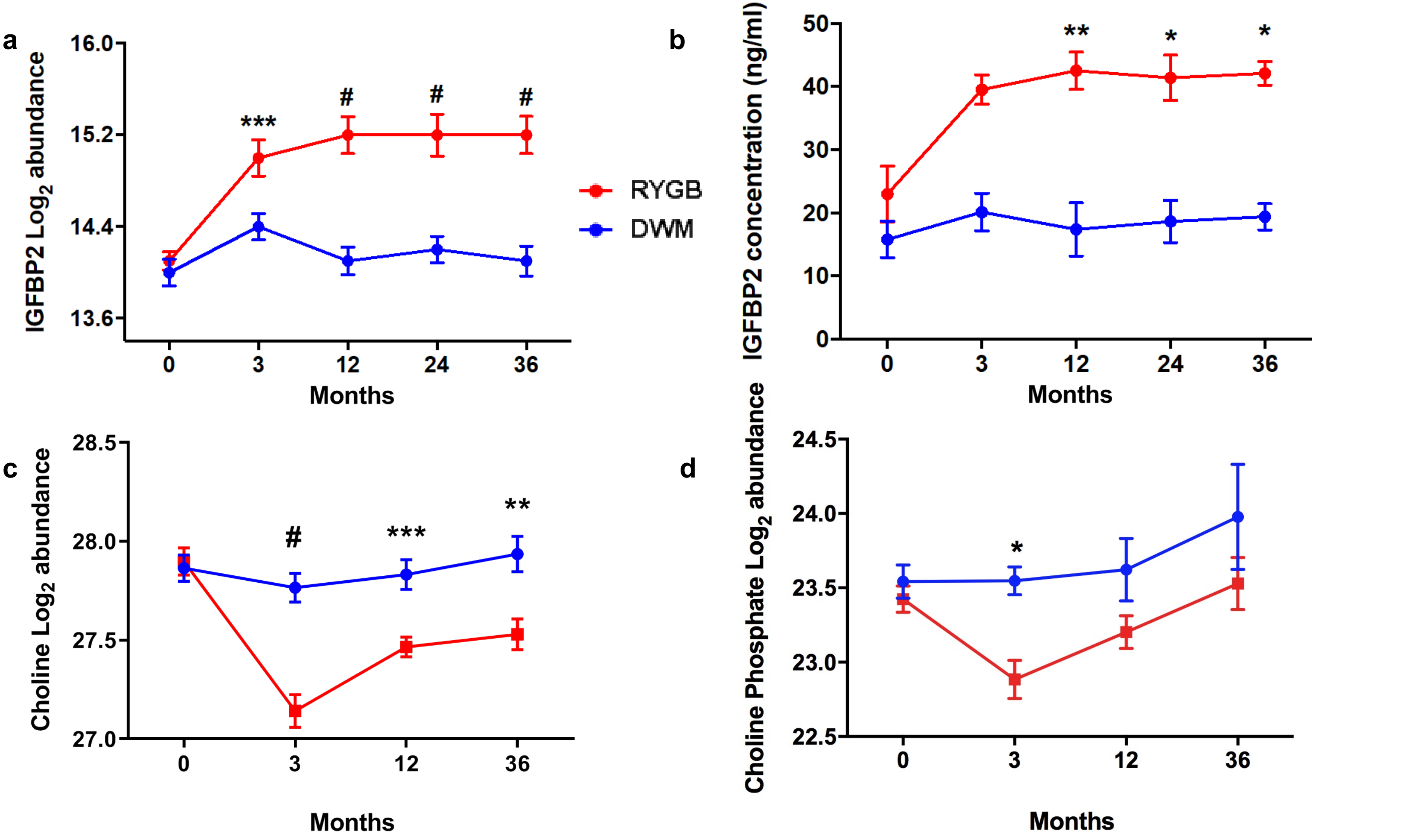

Figure S2. IGFBP2, choline, and choline phosphate levels. (a) IGFBP2 log_2 abundance measured by SOMAscan in samples from independent human participants at baseline, 3, 12, 24, and 36 months from 19, 19, 16, 10, and 9 DWM and 19, 19,19, 15, and 14 RYGB participants. **(b)** IGFBP2 concentration measured by ELISA in 4 RYGB participants and 4 DWM participants at all time points. **(c-d)** Choline and choline phosphate log_2 abundance measured by metabolomics in samples from independent human participants at baseline, 3, 12, and 36 months from 19, 18, 16, and 9 DWM and 19, 18, 19, and 13 RYGB participants. Data were analyzed by two-sided moderated t-tests; post-baseline time points were baseline-corrected. All data are reported as mean ± SEM. P values are indicated as follows: **a**: IGFBP2 (SOMAscan): 3 mo: ***=0.00018; 12, 24, and 36 mo: #<0.0001; **b** IGFBP2 (ELISA): 12 mo: **=0.0058; 24 mo: *=0.026, 36 mo: *=0.042; **c** choline: 3 mo: #<0.0001; 12 mo: ***=0.00035; 36 mo: **=0.0016; **d** choline phosphate: 3 mo: *=0.02. These p-values are nominal for **(a), (c), (d).** Supplementary Data 2 includes FDRs and source data for **a, c** and **d;** source data for **b** are provided as a Source Data file.

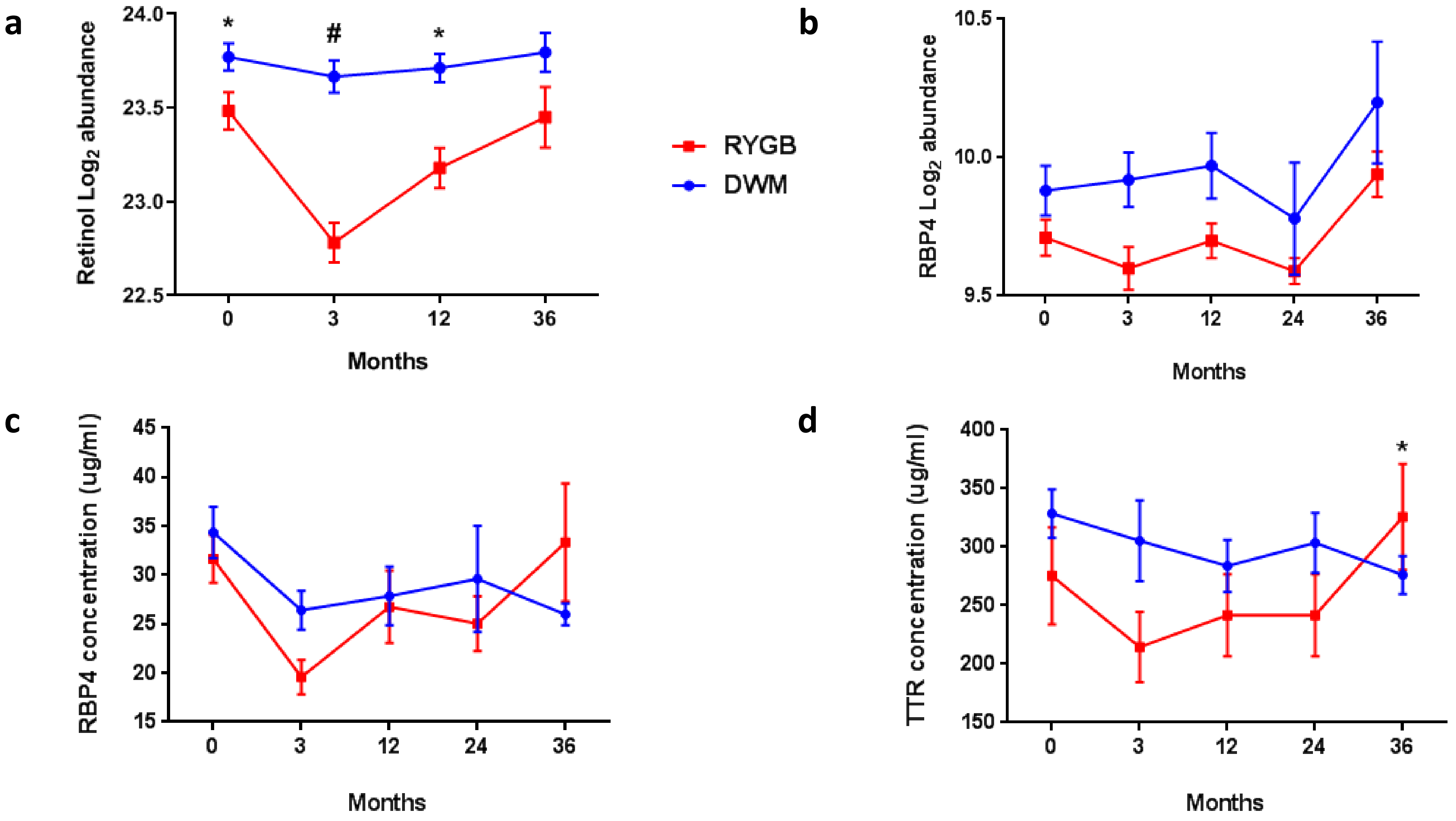

Figure S3. Retinol, RBP4, and TTR abundance. (a) Log_2 abundance of retinol measured by metabolomics in samples from independent human participants at baseline, 3, 12, and 36 months from 19, 18, 16, and 9 DWM and 19, 18, 19, and 13 RYGB participants. **(b)** Log_2 abundance of RBP4 measured by SOMAscan in samples from independent human participants at baseline, 3, 12, 24, and 36 months from 19, 19, 16, 10, and 9 DWM and 19, 19,19, 15, and 14 RYGB participants. **(c-d)** RBP4 and TTR plasma levels measured by quantitative western blot in 6 RYGB participants at baseline, 3, 12, 24, and 36 months, and 6 DWM participants at baseline, 3, 12, and 24 months but 4 participants at 36 months. Data were analyzed by moderated two-sided t-tests; post-baseline time points were baseline-corrected. Data are reported as mean ± SEM, and p values are nominal for **(a)** and **(b)**; FDRs are reported in Table S2. P values designated by symbols are as follows: (a) retinol: 0 mo: *=0.035; 3 mo: #<0.0001; 12 mo: *=0.018. (b) TTR: 36 mo: *=0.038. Source data for **a** and **b** are provided in Data S2, and source data for **c** and **d** are provided as a Source Data file.

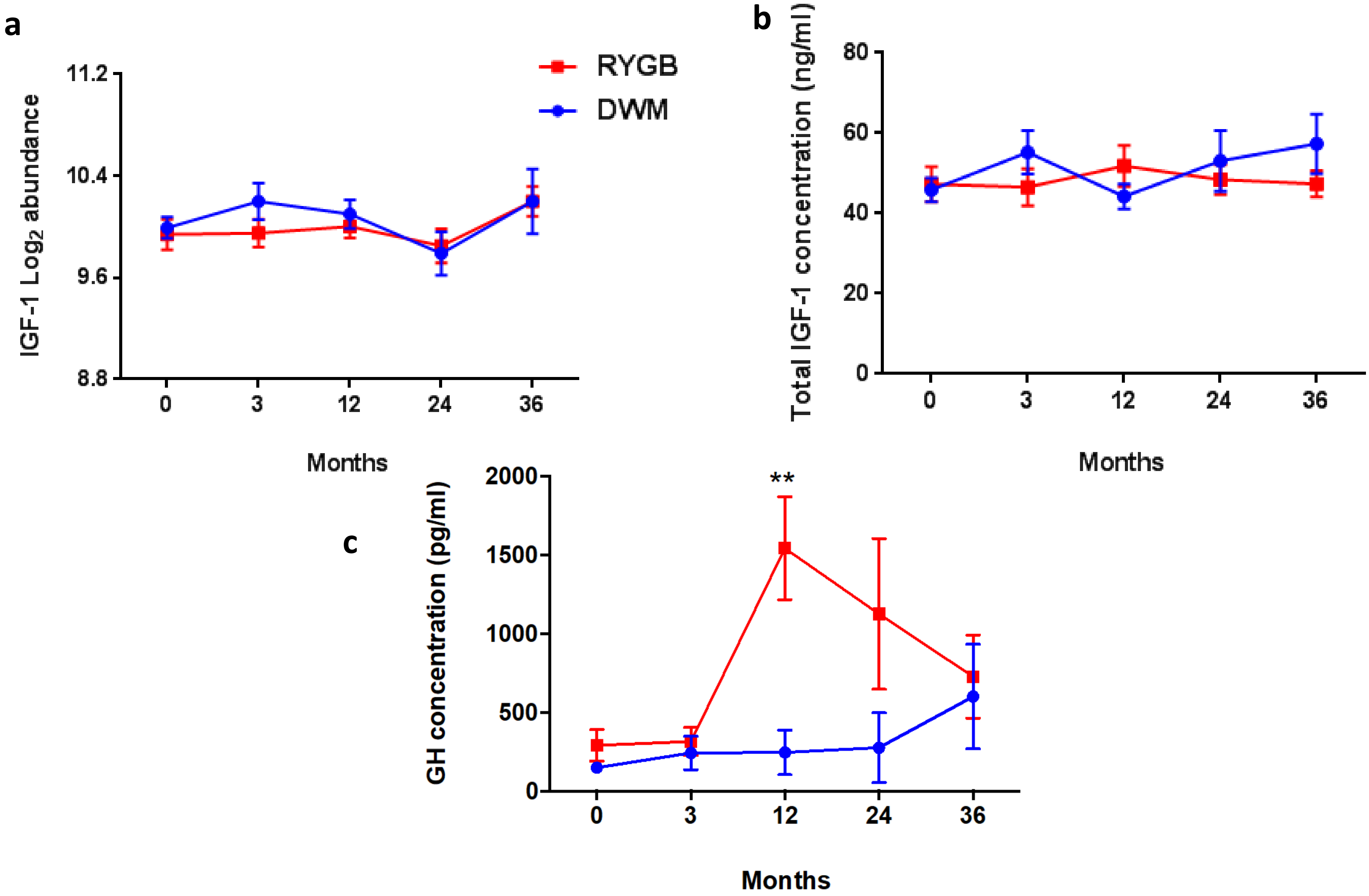

Figure S4. IGF-1 and GH abundance. (a) Log_2 abundance of IGF-1 measured by SOMAscan in samples from independent human participants at baseline, 3, 12, 24, and 36 months from 19, 19, 16, 10, and 9 DWM and 19, 19,19, 15, and 14 RYGB participants. **(b)** Plasma levels of total IGF-1 measured by ELISA at baseline, 3, 12, 24, and 36 months from 9, 9, 9, 7, and 7 RYGB participants and 9, 9, 9, 6, and 4 DWM participants, respectively. **(c)** Plasma levels of growth hormone measured by ELISA at baseline, 3, 12, 24, and 36 months from 11, 11, 11, 8, and 8 RYGB participants and 9, 9, 9, 6, and 4 DWM participants, respectively. Data were analyzed by moderated two-sided t-tests; post-baseline time points were baseline-corrected. Data are reported as mean ± SEM: **=0.0057. Source data and FDRs for **a** are provided in Data S2, and source data for **b** and **c** are provided as a Source Data file.

Supplementary Table 1: Primers

Primer	Primer Sequence (5' – 3')
Ghr_Forward	CTGCAAAGAATCAATCCAAGCC
Ghr_Reverse	CAGTTCAGGGGAACGACACT
Igf1_Forward	CACATCATGTCGTCTTCACACC
Igf1_Reverse	GGAAGCAACACTCATCCACAATG
Pepck_Forward	CTAACTTGGCCATGATGAACC
Pepck_Reverse	CTTCACTGAGGTGCCAGGAG
G6pc_Forward	TCTTGTCAGGCATTGCTGTGGC
G6pc_Reverse	GGTGGACCCATTCTGGCCGC
Socs1_Forward	CTGCGGCTTCTATTGGGGAC
Socs1_Reverse	AAAAGGCAGTCGAAGGTCTCG
Socs2_Forward	AGTTCGCATTCAGACTACCTACT
Socs2_Reverse	TGGTACTCAATCCGCAGGTTAG

References

1. Pearl, J. An introduction to causal inference. *Int J Biostat* **6**, Article 7 (2010).

2. Pearl, J. Comment: Understanding simpsons paradox. *The American Statistician* **68**, 8–13 (2014).

3. Judd, C. M. & Kenny, D. A. Process analysis. *Eval Rev* **5**, 602–619 (1981).

4. MacKinnon, D. P., Lockwood, C. M., Hoffman, J. M., West, S. G. & Sheets, V. A comparison of methods to test mediation and other intervening variable effects. *Psychol Methods* **7**, 83–104 (2002).

5. Huang, C.-C. *et al.* Activity dependent mammalian target of rapamycin pathway and brain derived neurotrophic factor release is required for the rapid antidepressant effects of puerarin. *Am J Chin Med* 1–16 (2018).

6. Ritchie, M. E. *et al.* Limma powers differential expression analyses for RNA-sequencing and microarray studies. *Nucleic Acids Res* **43**, e47 (2015).

7. Law, C. W., Chen, Y., Shi, W. & Smyth, G. K. Voom: Precision weights unlock linear model analysis tools for RNA-seq read counts. *Genome Biol* **15**, R29 (2014).

8. Smyth, G. K. Linear models and empirical bayes methods for assessing differential expression in microarray experiments. *Stat Appl Genet Mol Biol* **3**, Article3 (2004).

9. Djordjilović, V. *et al.* Global test for high-dimensional mediation: Testing groups of potential mediators. *Stat Med* **38**, 3346–3360 (2019).

10. Pearl, J. The deductive approach to causal inference. *J Causal Inference* **2**, (2014).

11. Barfield, R. *et al.* Testing for the indirect effect under the null for genome-wide mediation analyses. *Genet Epidemiol* **41**, 824–833 (2017).

12. Robinson, M. D. & Oshlack, A. A scaling normalization method for differential expression analysis of RNA-seq data. *Genome Biol* **11**, R25 (2010).

13. Oliva, M. *et al.* The impact of sex on gene expression across human tissues. *Science* **369**, (2020).
