# Supplementary material for: High-throughput mediation analysis of human proteome and metabolome identifies mediators of post-bariatric surgical diabetes control
Source: Nat Commun. 2021 Nov 29;12:6951. doi: 10.1038/s41467-021-27289-2 (PMC8630169; doi:10.1038/s41467-021-27289-2)
Supplement: Supplementary file 2 — Description of Additional Supplementary Files [file 41467_2021_27289_MOESM2_ESM.docx]

**Supplementary Data 1. Clinical and demographic tables.** Summary of the metabolic and demographic characteristics of the subset of participants who had omics data vs. the entire cohort.

**Supplementary Data 2. Differential abundance, correlation, and mediation tables.** Differential abundance of proteins, metabolites, and pathways; Pearson correlation coefficients of top proteins vs. top metabolites; mediation of HbA1c, insulin secretion (as the change in insulin from 0 to 30 minutes during a mixed meal tolerance test), and HOMA-IR.

**Supplementary Data 3. Metabolomics data from Metabolon.** Metabolite annotation at left, brief phenotype annotation at top, and the abundances on the original scale, i.e. unlogged and non-imputed.
